# A Review of Telomere Attrition in Cancer and Aging: Current Molecular Insights and Future Therapeutic Approaches

**DOI:** 10.3390/cancers17020257

**Published:** 2025-01-14

**Authors:** Mina Iskandar, Miguel Xiao Barbero, Muhamed Jaber, Roy Chen, Romulo Gomez-Guevara, Edwin Cruz, Sandy Westerheide

**Affiliations:** Department of Molecular Biosciences, University of South Florida, 4202 East Fowler Avenue, ISA2015, Tampa, FL 33620, USA; minaiskandar@usf.edu (M.I.); miguelxiaobarbero@usf.edu (M.X.B.); jaberm@usf.edu (M.J.); chenr@usf.edu (R.C.); romulo2@usf.edu (R.G.-G.); ecruz12@usf.edu (E.C.)

**Keywords:** telomere attrition, aging, cancer, telomerase reactivation, alternative lengthening of telomere, premature aging disorders, age-related diseases, therapeutics, biomarkers

## Abstract

Background/Objectives: As cells divide, telomeres shorten through a phenomenon known as telomere attrition, which leads to unavoidable senescence of cells. Unprotected DNA exponentially increases the odds of mutations, which can evolve into premature aging disorders and tumorigenesis. There has been growing academic and clinical interest in exploring this duality and developing optimal therapeutic strategies to combat telomere attrition in aging and cellular immortality in cancer. The purpose of this review is to provide an updated overview of telomere biology and therapeutic tactics to address aging and cancer. Methods: We used the Rayyan platform to review the PubMed database and examined the ClinicalTrial.gov registry to gain insight into clinical trials and their results. Results: Cancer cells activate telomerase or utilize alternative lengthening of telomeres to escape telomere shortening, leading to near immortality. Contrarily, normal cells experience telomeric erosion, contributing to premature aging disorders, such as Werner syndrome and Hutchinson–Gilford Progeria, and (2) aging-related diseases, such as neurodegenerative and cardiovascular diseases. Conclusions: The literature presents several promising therapeutic approaches to potentially balance telomere maintenance in aging and shortening in cancer. This review highlights gaps in knowledge and points to the potential of these optimal interventions in preclinical and clinical studies to inform future research in cancer and aging.

## Simple Summary

Telomeres play an integral role in preventing genomic instability by protecting chromosomal ends using telomeric repeats, and their dysfunction may lead to premature aging disorders and age-related diseases. During cellular division, they undergo telomere attrition, resulting in the gradual shortening of those protective caps, leading to genomic instability and cellular aging. Critically short telomeres in normal cells trigger senescence, a major contributor to age-related diseases, and opens the door for genomic instability, which promotes carcinogenesis. This review aims to provide an updated overview of telomere biology and therapeutic strategies around telomere attrition. We emphasize the importance of understanding the complex balance between preserving telomere length in aging while inhibiting telomere maintenance in cancer by proposing Optimal Therapeutic Approaches based on the most updated literature.

## 1. Introduction

### 1.1. Structure and Maintenance of Telomeres

**Figure 1:**
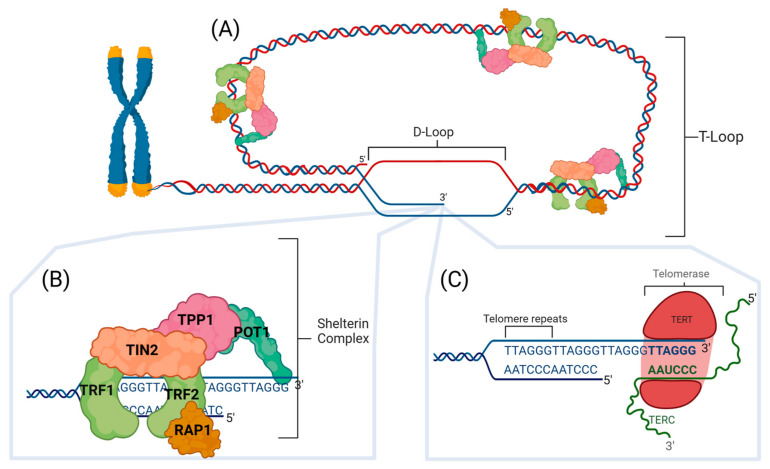
Shelterin complex and telomerase structures. (**A**) The T-loop structure found at the end of the chromosome contains the displacement loop or D-loop, where the shelterin complex protects the 3′ overhang when bound to the double strand. (**B**) The structure of the human shelterin complex is comprised of TPP1, POT1, TIN2, TRF1, TRF2, and RAP 1 subunits, which are responsible for protecting the telomere from the DDR. (**C**) Telomerase, consisting of human telomerase reverse transcriptase (hTERT) and human telomerase RNA component (hTERC) subunits, binds to telomeres at the 3′ overhang region during the late S-phase of the cell cycle. hTERC’s RNA template region anneals several of its starting nucleotides to the distal nucleotides of the overhang, and a new telomere repeat is added through hTERT, thus elongating the telomere. Figure colors are used for contrast only and do not represent any meaning beyond showing the different elements of the figure.

Mammalian chromosomal ends are represented by telomeres, which are 5–15 kilobases of repeated nucleotide sequences (5′-(TTAAGGG)n-3′) [1,2,3,4,5] that terminate in a single-stranded G-rich 3′ overhang [6] of 50–400 nucleotides [7,8]. As a result of semi-conservative DNA replication [9], the 3′ overhang is found at the end of the leading strand [10,11], and the lagging strand is partially replicated due to the removal of an RNA primer, which lies near the terminal end of the telomere [12,13]. The 3′ overhang, which must be at least six nucleotides in length [14], weaves back into the duplex telomeric strands and intercalates to form the D-loop, while the double-stranded telomere forms the T-loop (see Figure 1A) [15,16,17]. The T-loop structure is formed from this strand invasion, and it creates a lariat with the D-loop that helps the cell to distinguish between chromosome ends and actual sites of DNA damage [14]. Partial DNA replication causes the telomere to shorten by roughly 70–200 bases per division [14]; therefore, the loss of the telomeric DNA can cause structural damage to the T-loop. Shelterin proteins are also crucial for maintaining and stabilizing the T-loop [18]. When these proteins can no longer bind to the telomere, the protective structure is lost, exposing the ends [19]. Uncapped ends of telomeres are now recognized as double-strand breaks (DSBs) [20], which, if repaired, may give rise to a fusion of chromosomes and a cascade of genetic instability from breakage fusion cycles following mitosis [21].

The shelterin complex helps stabilize the T-loop and regulate the maintenance of telomere length [22,23,24]. As displayed in Figure 1B, this protective structure consists of the TRF1, TRF2, TIN2, POT1, TPP1, and RAP1 subunits [18,24]. TRF1 and TRF2 are considered the foundation of the complex by fostering telomere replication and recognizing the dsDNA [25,26]. The role of TIN2 is to serve as a binding protein between all the shelterin proteins [25,27]. POT1 is responsible for binding to the 3′ overhang [27], while RAP1 locates the telomere, serving as a guide for TRF2 [28]. Lastly, TPP1 plays a defensive role, promoting telomerase recruitment [29]; the function of TPP1 was highlighted in research where mutations in TPP1 resulted in DDR activation, leading to telomere hyperextension and telomere uncapping [30,31,32].

Apart from T-loops and the shelterin complex, cells have many other mechanisms to maintain telomere length and structure, such as the use of telomerase. Telomerase activity is typically undetectable within most adult somatic cells due to tightly regulated TERT expression following differentiation [33,34,35]; however, it is abundant in certain highly proliferative cells like neoplastic, stem, and germ line cells [35,36,37]. Also, lymphocytes, like T and B cells, show readily detectable telomerase activity upon stimulation [33,38]. In comparison, cancer cells exhibit constitutive telomerase activity, though not as strictly regulated as normal and stem cells [39]. Thus, telomerase regulation plays an important role in these cells to ensure that their highly replicative nature does not result in uncapped telomeres and, therefore, genomic instability. Telomerase maintains telomere length through the addition of nucleotides, and it is made up of two main subunits: human telomerase reverse transcriptase (hTERT) and human telomerase RNA component (hTERC, also known as hTR) (see Figure 1C) [40]. hTERT is the catalytic subunit, which is significantly expressed in neoplastic, stem, and germ line cells [41]; hTERC is expressed in almost all cells [35,42]. The telomerase RNA gene hTERC contains the CAAUCCCAAUC sequence, which serves as the template region for telomere replication [43]. This sequence only represents 11 nucleotides out of the 451 nucleotides, while the remaining act as domains for various functions [43].

### 1.2. Significance of Telomere Attrition in Cancer and Aging

**Figure 2:**
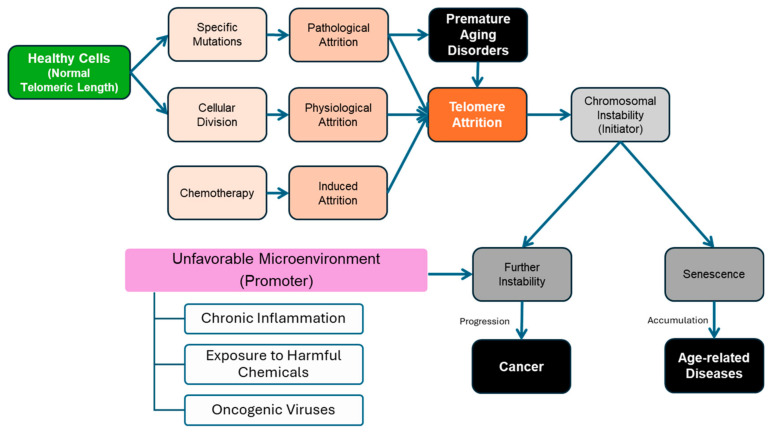
Progression of healthy cells into cancer, age-related diseases, and premature aging disorders. Telomere attrition can mainly occur through physiological, pathological (which leads to premature aging disorders), and chemotherapy-induced attrition. Chromosomal instability coupled with a damaging proliferative microenvironment can lead to cancer progression or generation of senescent cells, which, if accumulated, can lead to age-related diseases. Figure colors are used for contrast only and do not represent any meaning beyond showing the different elements of the figure.

Due to the lack of telomerase expression, the telomeres of somatic cells that are not terminally differentiated erode with every replication cycle (physiological attrition, see Figure 2). Such incomplete replication is described as the End Replication Problem [44,45]. ERP is the main mechanism driving telomere shortening, losing around 50–200 base pairs per division [46,47]. Eventually, cells reach the Hayflick Limit, which is the finite number of population doublings a somatic cell can undergo in culture [46]. They can no longer replicate without causing catastrophic damage to their genome; therefore, the cells induce senescence to ensure the termination of replication [45,46,48]. It is important to note that senescence is the state of irreversible cell arrest, which occurs within mitotic cells that are still metabolically active. Aside from replicative senescence, which is telomere-dependent, stress-induced premature senescence (SIPS) may occur independent of telomere attrition [49]. Cells can rely on pathways like Rb and p53 [50,51] to activate premature senescence by silencing the transcription factor E2F and its downstream target genes, which are integral for the continuation of the cell cycle [52,53]. Thus, senescence is a tumor suppressor mechanism that can prevent catastrophic genomic instability, which might lead to cancer [54,55].

However, as we age, these senescent cells accumulate, which may contribute to the development of age-related diseases, such as cardiovascular, neurodegeneration, and other diseases [56]. For example, such senescent cells secrete a plethora of pro-inflammatory proteins, causing chronic inflammation and endothelial dysfunction. This chronic inflammation may then result in several pathologies, such as atherosclerosis, heart failure, and cardiac remodeling [57,58]. Additionally, although senescence can act as a barrier to tumorigenesis [59,60], it is often accompanied by (1) telomeric dysfunction, leading to genomic instability [51,61,62], and (2) the secretion of cytokines, proteases, and growth factors, resulting in chronic inflammation and oxidative microenvironment, which contribute to the initiation and promotion of carcinogenesis (see Figure 2) [63,64]. Some individuals may have mutations within the components of the shelterin complex or telomerase. The resulting defects from these mutations may cause premature aging disorders [65] that are associated with accelerated telomeric shortening and increased likelihood of developing cancer, such as Werner syndrome [66,67,68], Bloom syndrome [69], and dyskeratosis congenita [70,71].

Not only does telomere attrition result in vulnerable genetic material, leaving cells prone to carcinogenesis, but it is also the biggest obstacle cancer faces during its development. Like healthy cells, the telomeres in tumor cells become so short that replication is impaired, reducing the cancer’s proliferative capacity. In most cases, cancer cells bypass this hurdle through telomerase activation, promoting the telomere elongation mechanism [37,72,73]. However, some types of cancer cells do not activate telomerase and resort to a different mechanism dubbed the alternative lengthening of telomeres (ALT), where homologous recombination and sister chromatid exchange are used to restore the telomere length [74,75,76,77]. ALT can occur when cancers are treated with telomerase inhibitors, causing selective pressure for resistance, and they develop ALT to continue maintaining their telomere length even while telomerase is inhibited [78]. This paper will touch on the intricacies of the various mechanisms that fall under the umbrella term of ALT, as well as potential therapeutic targets to avoid chemotherapy resistance.

### 1.3. Review Objectives: Examine the Biology of Telomeres and Therapeutic Approaches

Because of the duality of telomere biology in cancer and aging, there has been a growing academic and clinical interest. This paper aims to present an updated review of the current state of telomere physiology along with potential therapeutics to inform future research in cancer and aging. Effective therapeutic approaches require careful interventions to balance the induction of telomere erosion to treat cancer and telomere maintenance to halt aging. This duality within telomere maintenance emphasizes the necessity for refined therapeutic strategies. Treatments aimed at halting telomere elongation within cancer must not accelerate aging (induced attrition), while interventions that seek to preserve telomeres must consider the possibility of increased cancer risk. Thus, forming a strict harmony between maintaining and shortening telomeres is critical in attaining healthy aging and cancer treatment and the Optimal Therapeutic Approach (OTA). The literature reveals many promising pharmacological compounds, genetic approaches, and natural strategies to achieve this balance. Moreover, identifying reliable biomarkers linked to specific telomere-related disorders is essential for achieving OTA and enabling early detection to improve clinical outcomes.

## 2. Methods

### 2.1. PubMed Database and Rayyan Platform

**Figure 3:**
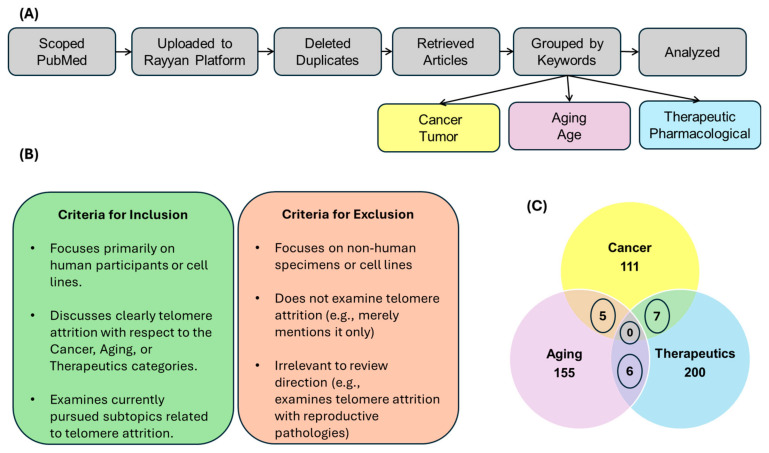
Review progression and scoping criteria. (**A**) Articles were scoped from PubMed using subtopics related to telomere attrition examined in the review. The selection was uploaded to the Rayyan platform and checked for duplicates. Articles were retrieved and grouped based on these keywords. (**B**) The criteria used to include or exclude papers in the review. (**C**) Total articles retrieved and analyzed in each category.

We searched the PubMed database for articles that focus on telomere attrition with respect to cancer, aging, and therapeutics. We used the criteria for inclusion and exclusion, depicted in Figure 3B, during our scoping phase to gather relevant articles for this review’s objectives. The search results were uploaded to the Rayyan legacy platform, where they were automatically checked for duplicates. The remaining articles were grouped into three main categories, as shown in Figure 3A. In total, we reviewed 466 papers: 111 predominantly focusing on Cancer, 155 on Aging, and 200 on Therapeutics (Figure 3C). All open-access papers were downloaded directly from PubMed, while subscription publications were requested and downloaded through USF Libraries ILLiad. All data analyses were performed using Microsoft Excel, and all figures were created using Microsoft PowerPoint or BioRender.

### 2.2. ClinicalTrial.gov Registry

Most of the papers analyzed included preclinical studies, and some articles mentioned drugs in clinical trials; however, even though the PubMed database was updated, it did not contain updates on the progress of these preclinical studies or clinical trials. To overcome this limitation, we utilized the ClinicalTrial.gov website (accessed from 1 October 2024 to 10 October 2024) to gain a more comprehensive update (see Supplementary File S4). We included trials that started during or after 2014 to present a more recent clinical perspective on these reported drugs. Additionally, we examined trials that focused on the drug of interest as the primary intervention or in combination with first-line treatments. We considered clinical trials of all phases and statuses but limited the search to conditions connected to “cancer”, “aging”, and “telomeres”. To gain a broad overview, we considered trials of all funding types and did not filter trials based on age, sex, or outcome measured.

## 3. Telomere Attrition in Cancer

### 3.1. Telomere Attrition Drives Genomic Instability

Telomeres play a crucial role in maintaining genomic stability, and their dysfunction is intricately linked to the development and progression of cancer. As telomeres reach a critical length, they become vulnerable to genetic instability; cells recognize this and stop it by inducing senescence or apoptosis. Senescence and apoptosis are the cells’ most important defenses against carcinogenic mutations [79,80,81], yet cells have two main proposed ways to bypass this protection. First, cells with critically short telomeres that become senescent remain metabolically active and express specific genes, which results in the release of pro-inflammatory cytokines into the microenvironment, creating carcinogenic surroundings for nearby cells [82,83]. Second, cells with critically short telomeres may evade senescence and apoptosis by inactivating tumor suppressors like p53 [84]. This bypasses cell cycle checkpoints and, with ALT or telomerase reactivation, leads to the uncontrolled proliferation characteristic of cancer [76,85].

Before becoming malignant, cancer cells must acquire a multitude of mutations, where a series of at least 20 to 30 cell divisions is necessary for every mutation [86]. It is proposed that the cell that harbors that first mutation must replicate into roughly one million cells to increase the likelihood for a second mutation to take place [86]. Over time, these mutations may include the loss of p53 or Rb function, allowing the pre-cancerous cell to bypass the M1 stage, the point where critical telomere length is reached, and replicative senescence follows the many rounds of replication [87].

Telomeres continue to shorten with each division as certain cell cycle checkpoints are inactivated. As cells progress to the M2 stage, referred to as ‘crisis’, they exhibit a large presence of vulnerable and critically short telomeres, apoptotic activity, and mitotic stress [87,88]. Through telomerase reactivation [89,90,91] and the alternative lengthening of telomeres (ALT) [92,93], these precancerous cells may now escape the crisis stage through telomere maintenance [86]. This results in indefinite proliferation, which may contribute to carcinogenesis [86] if coupled with mutations in oncogenes and tumor suppressor genes [94].

### 3.2. Dysregulation of Telomerase Activity

**Figure 4:**
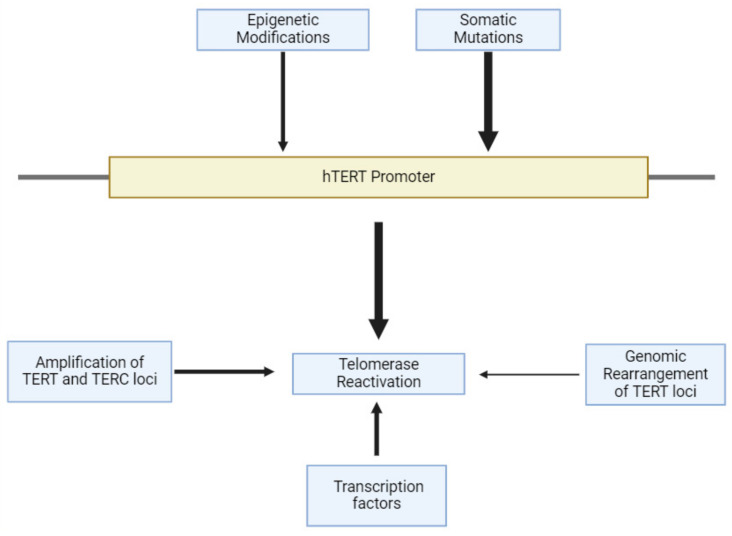
Mechanisms for telomerase reactivation. Telomerase reactivation can occur through five main mechanisms: (1) amplification of TERT and TERC loci, (2) genomic Rearrangement of TERT loci, (3) epigenetic modification, (4) somatic mutations, and (5) transcription factors, with the last three directly affecting the hTERT promoter. The width of the arrow corresponds to the frequency of the mechanism.

Telomerase reactivation can occur through four main mechanisms: (1) somatic mutations within the TERT promoter (pTERT) [95], (2) epigenetic modifications of pTERT [96,97], (3) amplification of TERT and TERC [98,99,100], and (4) genomic rearrangement of TERT (see Figure 4) [101,102]. Most of the observed cancers that are telomerase-positive show somatic mutations within the pTERT, which leads to its activation [95,103]. In addition to p53 mutations [104,105], FOXO3 inhibition [106] and other genetic mutations can help increase the likelihood of pTERT activation; these generally occur within the ~124 and ~146 bp from the ATG start site of the pTERT, generating an increase in promoter activity and hTERT expression [107]. Moreover, mutations of genes related to the shelterin complex can increase the risk of cancer development [108,109,110], like the *POT1* gene mutation, which leads to the development of brain tumors [111].

Epigenetic modifications through pTERT methylation occur in a region extending from ~650 bp to ~217 bp of the ATG start site containing 52 CpG sites known as the TERT hypermethylated oncological regions (THOR) [112]. Methylation in this region is usually overexpressed in malignant tumors and under-expressed in normal tissues and stem cells [112,113]. The importance of epigenetic modifications in these areas of pTERT lies in that these regions bind many repressors, and once they are hypermethylated, the repression is halted, leading to an increase in the expression of telomerase [114].

While genetic mutations can initiate cancer, certain genes with higher levels of activity can increase the severity of cancer. A 2020 systematic analysis using 31 different tumor types from 6835 patients reported that TERT amplification is present within 4% of tumors [103]. Specifically, two loci—5p15.33 of TERT and 3q26.3 of TERC—are observed to be the most common amplifications within cancer types [115,116]. Some cancers with this TERT enhancement may include ovarian cancer, squamous-cell carcinoma, esophageal cancer, adrenocortical carcinoma, and lung adenocarcinoma [103]. As shown in one study on mouse oocytes, a translationally controlled tumor protein called TCTP, which aids in monitoring basic cellular processes, can increase telomerase activity if overexpressed [117]. In fact, TCTP is frequently overexpressed in cancer, indicating an increase in telomerase activity [117].

Genomic rearrangement of TERT is one of the most uncommon types of telomerase reactivation. Alterations adjacent to the locus 5p15.33, which contains TERT [102], cluster around a region located roughly 50 kb away from the TERT promoter site. These alterations activate super-enhancers and consequent chromatin remodeling, resulting in TERT overexpression [118,119]. This type of activation of telomerase is most common in high-risk neuroblastomas and is mutually exclusive to MYCN amplifications and ATRX mutations [102,119]. Various transcription factors, including c-Myc, NF-kB, Sp1, and STAT3, play an integral role in hTERT regulation [120]. More specifically, the hTERT promoter has two E-boxes, which allow for the precise binding of the heterodimer that forms between the transcriptional factors Max and c-Myc [120,121]. The bound heterodimer activates hTERT transcription, and a study from 2012 emphasizes the presence of a positive correlation between upregulated c-Myc and TERT expression within gastric precancerous lesions [122]. NF-kB upregulation also contributes to the downstream activation of hTERT through c-Myc, which is a favorable downstream effector of the NF-kB signaling pathway [123]. c-Myc has been considered a potential therapeutic target due to its association with disease progression and poor prognosis, which is a result of its role in hTERT promoter activation, amongst its other oncoprotein functions.

Telomerase reactivation is associated with a variety of cancer types. Specifically, one study found telomerase to be a strong predictor of survival outcomes for patients with breast cancer [124], non-small-cell lung cancer [125,126], and colorectal cancer [127,128]. Despite the sample size being too small for conclusive findings, patients with melanoma appear to have a relationship between telomerase activity and cancer severity [129,130,131]. Besides survival rates, hTERT promoter mutations could be a more sensitive biomarker for urinary bladder cancer diagnosis than urine cytology, as samples of this can be found in the urine of patients up to 10 years before the diagnosis of urinary bladder cancer [132]. Similarly, mesothelioma patients are found to have higher hTERT protein levels when their cancers are malignant, allowing clinicians to distinguish the severity of their patients’ disease [133]. Upregulation of transcriptional factors like c-Myc has been shown to increase hTERT expression in gastric precancerous lesions [122], highlighting the importance of continued investigation of transcriptional factors as hTERT regulators. Overall, these findings underscore the multifaceted role of telomerase reactivation in cancer progression, with varying genetic, epigenetic, and structural modifications driving its dysregulation across different malignancies. Understanding hTERT-promoting mechanisms in greater depth enhances our knowledge of cancer biology with regard to the indefinite proliferation of cells and exposes potential therapeutic targets for better cancer treatments.

### 3.3. Alternative Lengthening of Telomeres

#### 3.3.1. Homologous Recombination in ALT

**Figure 5:**
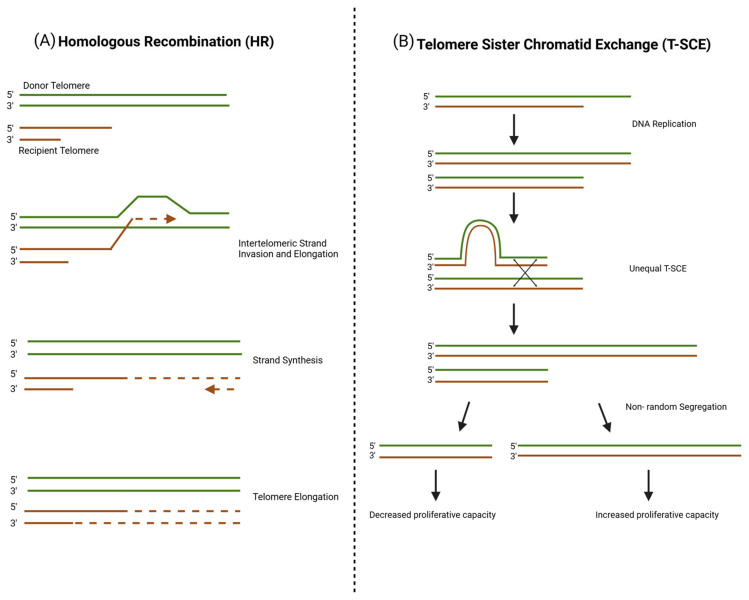
Alternative lengthening of telomeres (ALT) mechanisms. (**A**) Homologous recombination (HR) occurs when a recipient telomere uses a donor telomere as a template for telomere elongation. (**B**) Telomere sister chromatid exchange (T-SCE) is a process where sister chromatids exchange telomeric content. This exchange results in telomeres of varying lengths, contributing to the heterogeneous telomere lengths within the ALT-positive cell. With cell division, the daughter cell that acquires the shorter telomere may have reduced proliferative capacity, while the cell that acquires the lengthened form of the already short telomere may have extended proliferative capacity.

Homologous recombination (HR) is a commonly proposed ALT mechanism that results from recombination-mediated synthesis of new telomeric DNA. As seen in Figure 5A, the new telomeric DNA uses an existing telomere sequence from an adjacent chromosome’s telomere to copy from [134,135]. This model does not necessarily rely on using template DNA from the telomere of another chromosome, though it has been shown that the telomere of a non-homologous chromosome can serve as a template under certain conditions [75]. Homologous recombination is a well-understood pathway in the context of double-strand break repair; however, in the context of cancer cells, this recombination is dysregulated and hijacked to maintain telomere length and sustain proliferative capacity. Homologous recombination is an intricate pathway requiring the activation of transcription factors like PAX and SOX, which are activated by the TGF-β superfamily genes [136]. PAX and SOX activate RAD9B, RAD52, and many other proteins that are instrumental in establishing the foundation for homologous recombination [136].

Key components of the HR pathway are Rad51 and Rad52 [137,138,139,140]. Due to the conserved homologous recombination mechanism between yeast and mammalian cells, researchers often use yeast, Saccharomyces cerevisiae, as a model organism to study the intricate mechanism, including the specific roles of proteins like Rad51 and Rad52 [134]. In yeast model organisms, there were two types of ALT cells: (1) Type I, which is Rad51-dependent-Rad52-dependent, and (2) Type II, which is Rad52-dependent-Rad51-independent. Type I maintained telomeres by increasing the frequency of recurrent sub-telomeric sequences, while Type II maintained expansion of telomeric repeats [138]. Rad51 was determined to be the key recombinase in the HR pathway, which relies on Rad52. Rad52 binds Rad51 to single-stranded DNA and can anneal complementary ssDNA [141,142].

In ALT-positive cells, a defect in telomere capping, potentially linked to TRF2 dysfunction, may lead to failure to suppress telomeric HR, which could be essential for the ALT mechanism [143,144]. TRF2 may be capable of HR suppression through its protective role in T-loop and four-strand DNA junction synthesis [143,144]. Both facts suggest that TRF2 may have a role in regulating telomere recombination [145]. Altogether, the involvement of HR in the ALT mechanism, particularly its reliance on factors like Rad51 and Rad52, underscores the intricate dynamics of telomere maintenance in cancer cells. The observed enhancement of HR following telomerase inhibition suggests potential avenues for targeting ALT-positive tumors.

#### 3.3.2. Telomere Sister Chromatid Exchange (T-SCE)

Research shows that telomere lengthening in ALT cells relies on DNA recombination, with telomere sister chromatid exchange (T-SCE) playing a key role in this process [146]. T-SCE is a submechanism of homologous recombination (HR) and helps manage telomeric regions where structural gaps in the DNA create obstacles during replication [75,93,147]. T-SCE results in sister chromatids with telomeres of unequal lengths within the same ALT-positive cell [148]. Upon division, the daughter cell that inherits shorter telomeres may have reduced proliferative capacity and be eventually eliminated by selective pressures [149]. The daughter cell, which acquires longer telomeres from the exchange, may have more successful, continued proliferation [149] (see Figure 5B).

#### 3.3.3. ALT-Positive vs. ALT-Negative Phenotypes in Tumors

Many ALT-positive tumors have increased sensitivity to DNA damage and have slow DNA repair capabilities alongside defective G2/M checkpoints [150]. One major hallmark is ALT-associated PML bodies (APBs), which contain telomeric DNA and telomere-associated proteins/DNA repair factors [151,152,153]. They are found in greater abundance in tumors with microsatellite instability compared to microsatellite-stable tumors [154,155]. Additional hallmarks include heterogeneous telomeres, extrachromosomal telomeric DNA (i.e., c-circle DNA), heightened levels of spontaneous DNA damage localized to the telomeres, altered expression or mutations within the chromatin remodeler ATRX, and increased extrachromosomal telomere repeats (ECTR) [150,153,156,157]. Studies have elucidated variations in the insertions and repeats of telomeres at chromosomal ends across a wide range of ALT-positive cells [138].

With the existence of these ALT markers, a range of cancers that employ the ALT mechanism has been recognized, with many having a mesenchymal origin [158,159]. Alterations in the ATRX/DAXX complex and H3.3 histone have been identified as common mutations amongst ALT-positive cancers [150,160]. Mutations that lead to the loss of these genes have been seen to lead to a heterochromatin-repressed state, which activates chromosomal recombination, thus initiating ALT [161]. The loss of ATRX in ALT-positive cells could be an avenue for future therapeutic approaches, though it is important to note that the loss of ATRX alone is not enough to generate the ALT-positive phenotype [162]. A 2024 study concerning this inquiry elucidated that ALT-dependent pediatric high-grade glioma with mutations of ATRX and H3G34R, which are common within adolescents and youth, can respond very well to a poly-ADP ribose polymerase inhibitor (PARPi) combination therapy [163]. A combination of niraparib and topotecan was used to target Rad51 and PARylation levels in a 15-year-old female patient, which exploited deficiencies within the homologous recombination pathway and increased DNA replication stress in ALT-positive cells [163]. A substantial reduction in tumor size within the patient occurred; however, manifestation of a new tumor occurred roughly a year after combination treatment [163]. Thus, more research into ALT-dependent tumor treatments, potentially with larger patient cohorts, is necessary. This growing understanding of the molecular landscape of ALT tumors, especially those of mesenchymal origin, not only enhances diagnostic capabilities but also presents new opportunities for targeted therapeutic strategies in combating these aggressive cancer types.

Depletion of mediators such as RAD51 has been shown to diminish various known ALT hallmarks like T-SCE and ALT-associated PML bodies [164]. Another common association in ALT-positive cancer genomes that may be related to T-SCE is mutations in the ATRX/DAXX complex [150,157]. The prevalence of T-SCE within ALT cells highlights the adaptability of cancer cell mechanisms, where unequal T-SCE supports telomere length diversity and cellular survival [165]. The involvement of factors like RAD51 and the ATRX/DAXX complex suggests that further elaboration of this pathway could provide critical insights into how ALT-positive cancers maintain their proliferative capacity [162].

### 3.4. Telomere Maintenance Strategies: Telomerase Reactivation vs. ALT

**Figure 6:**
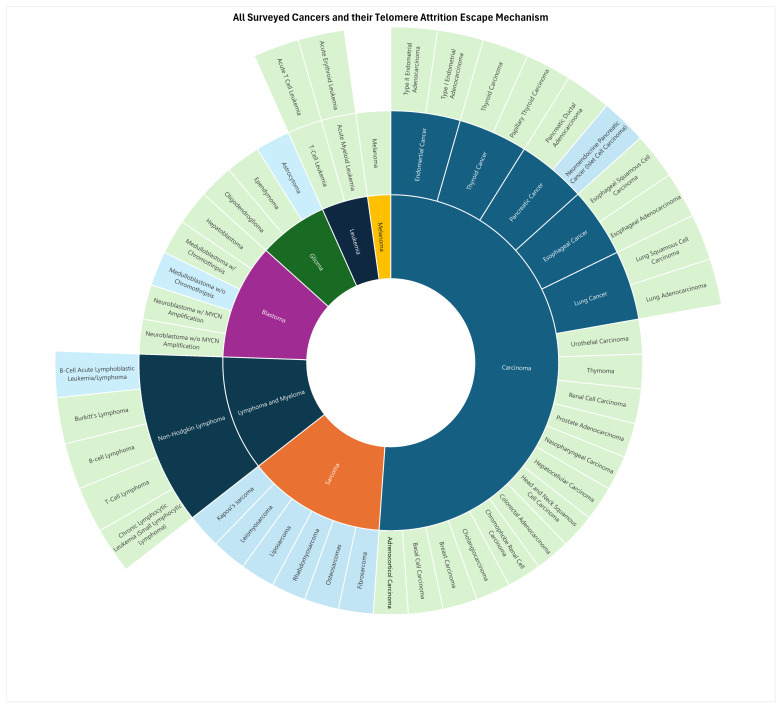
All surveyed cancers and their telomere attrition escape mechanisms. A sunburst diagram depicts 46 different types of cancer, their class, and their preferred mechanism of escaping telomere attrition, either telomerase reactivation (light green) or ALT (light blue) (see Supplementary File S1).

Understanding what types of cancers undergo ALT and telomerase reactivation is pivotal for guiding clinical treatment protocols. Our review wishes to consolidate data from the literature reviewed to provide a comprehensive overview, contributing to and supporting the field’s current understanding. As seen in Figure 6, we found that 10 out of 47 cancer types (21%) predominantly utilize ALT, while the remaining 79% favor telomerase reactivation (see Supplementary File S1). These data are relatively similar to the common notion that ~85% of cancers prefer telomerase reactivation, and ~15% prefer ALT [166]. Our findings highlight patterns in what types of cancer tend to prefer ALT rather than telomerase reactivation, with all sarcomas showing a preference for ALT, as seen in Figure 6. These data are reinforced by extensive amounts of literature that point toward sarcomas preferring ALT, and ALT-focused treatments show promise in treating these cancers [158,167,168].

### 3.5. Therapeutic Potential of Telomere Length and Telomerase in Cancer

Telomere length is commonly used as a biomarker for detecting cancer and predicting prognosis, as it is present in various cancer types. Long relative telomere length, measured via whole-genome sequencing and quantitative PCR, was associated with increased aggressiveness, low cell differentiation, poor survival outcomes, and high tumor proliferation in hepatocellular carcinoma patient samples [169]. In contrast, studies on breast cancer have shown that tumors with higher grades have shorter telomeres and higher levels of telomerase [170,171]. These findings demonstrate that telomere length is a promising sensitive biomarker for cancer progression. Additionally, it shows a potential to exploit this knowledge to develop telomere-targeted therapeutics.

Telomerase itself shows promising applications as a prognostic biomarker; patients with high hTERT expression were observed to have worse survival rates than those with low hTERT expression [172]. These findings are consistent with the importance of telomerase for cancer to overcome telomere attrition caused by its highly proliferative nature. As highlighted previously in Figure 2, telomere attrition can act as a gateway to chromosomal instability (initiator) that, if coupled with the damaging microenvironment (promoter), leads to carcinogenesis [172]. This observation was also made by the Dana–Farber Cancer Institute, where they suggested that tumor size and chromosomal instability play a crucial role in metastatic lymph node development [173]. Telomere attrition becomes an even bigger obstacle to immortality; thus, hTERT expression levels increase due to telomerase reactivation as tumor cells undergo natural selection to overcome telomere attrition.

ALT-positive cells have been targeted by therapies in a multitude of ways, including the use of CRSIPR-Cas9 to delete ALT pathway genes, knocking down genes pivotal for PML body synthesis, and even promoting ALT-specific lethality through ATR kinase inhibition [34,174]. That said, one of the biggest challenges for treating ALT-positive cancer cells is that most were once telomerase-activating cells that have shifted to ALT after encountering telomerase-inhibiting therapies [78]. The CRISPR-Cas9 deletion of ALT pathway genes is suggested as a future direction in a study conducted by researchers at the University of Michigan, where they highlight the efficacy of using CRISPR-Cas9 to delete telomerase genes as an anticancer therapy [34]. They propose a multifocal therapeutic strategy using CRISPR-Cas9, where one would target ALT pathway genes, telomerase genes, and other key cancer survival genes for a comprehensive therapy to combat resistance [34]. Knockdown of the ligase MMS21, which is critical for the synthesis of the APB bodies, has resulted in telomere shortening in ALT-positive cells [175]. These APB bodies aid in localizing the BLM–TOP3A–RMI (BTR) complex to the telomere ends of ALT-positive cells, which allows for c-circle formation and heterogenous telomere length maintenance [176]. A similar effect is also seen in TSPYL5 targeting therapies, with TSPYL5 being a protein that is specific to ALT-positive cells [177]. Inhibition of TSPYL5 results in massive amounts of cell death from degradation of POT1 [174]. ATR kinase inhibition has also shown promising results with regard to ALT-specific therapies, where ATR kinase is inhibited and can no longer regulate the cell’s stress response, making the telomeres more fragile and prone to DSB [178,179]. This process significantly hinders the ability of ALT-positive cells to replicate, leading to halted elongation of fractured telomeres [174] and even apoptosis of those ALT-positive cells [180]. All these therapeutic strategies highlight promising avenues for future directions with regard to targeting ALT-positive cells, and these therapies, in combination with telomerase inhibitors, may prove to be effective.

### 3.6. Chemotherapy and Its Effect on Telomeres

Chemotherapy, a pillar of cancer treatment, promotes a constant cycle of damage and repair within cancerous and normal cells. Cisplatin, a cytotoxic drug, has been shown to preferentially bind to the highly prevalent guanine–guanine and adenine–guanine repeats within telomeric DNA [181,182], contributing to an increase in telomere attrition by ~145-fold [183]. The DNA adducts formed at those specific repeats effectively prevent DNA replication, progression of the cell cycle, and associated processes that affect DNA, including those of telomeres [181]. Additionally, cisplatin has been shown to reduce antioxidant levels within the plasma, ultimately increasing oxidative damage and creating a damaging microenvironment [184,185,186]. This damage contributes to the higher exacerbation of age-related degeneration within elderly patients despite inducing telomere attrition in cancer cells [181]. Cisplatin is an example of a successful yet unbalanced therapeutic approach. Cisplatin helps treat patients’ cancer but increases their risk of age-related complications due to the increase in unselective telomere attrition and promotion of oxidative damage, two main factors that contribute to age-related diseases.

Other chemotherapeutic drugs have shown similar unbalanced effects, like arabinoside and daunorubicin, which interfere with DNA replication, inducing apoptosis [187]. These drugs also damage and stress normal cells, which accelerates and disrupts the normal aging processes [187]. Combination chemotherapies like CHOP (cyclophosphamide, doxorubicin, vincristine, and prednisone) and ESHAP (etoposide, cisplatin, cytarabine, and methylprednisolone) have shown promising results when combating cancer, but they unfortunately promote telomere attrition. Compared to control, CHOP increased telomere attrition within PBMCs by around 35% between 6 and 12 months, with telomeres staying shortened two years later [188], and patients that were given ESHAP after CHOP were found to have increased telomere attrition by 50% in only two months after the treatment [188]. This telomere attrition can promote the rise and accumulation of senescent cells and, consequently, promote aging-related diseases.

Oxidative damage plays a fundamental role in balancing the delicate variables needed when treating cancer with chemotherapy. Oxidative stress that is caused by disrupted redox balance from excessive ROS generation, alongside antioxidant defense weakening [189], overwhelms cellular antioxidant capacity [190]. Consequently, ROS can suppress telomerase function [191] and directly impact the aging process [192]. Oxidative stress may lead to the release of calcium into mitochondria, resulting in dysfunction within the organelle and accelerated telomere shortening [193]. Additionally, oxidative stress can induce the translocation of TERT from the nucleus to the mitochondria, where it mitigates ROS levels and modulates cellular sensitivity to DNA damage and apoptosis [194]. Such modulation of ROS levels can prevent nuclear damage and may be attributed to the strong stress resistance that is exhibited by cancer cells [194]. Thus, allowing abnormally high levels of oxidative stress may represent possibly beneficial chemotherapeutic features but also warrant a strong disregard for the accelerated effects of aging and cancer development. An Optimal Therapeutic Approach is needed to reduce accelerated aging and the potential for stress resistance development in cancer cells from chemotherapy drug use.

## 4. Optimal Therapeutic Approaches for Cancer

**Figure 7:**
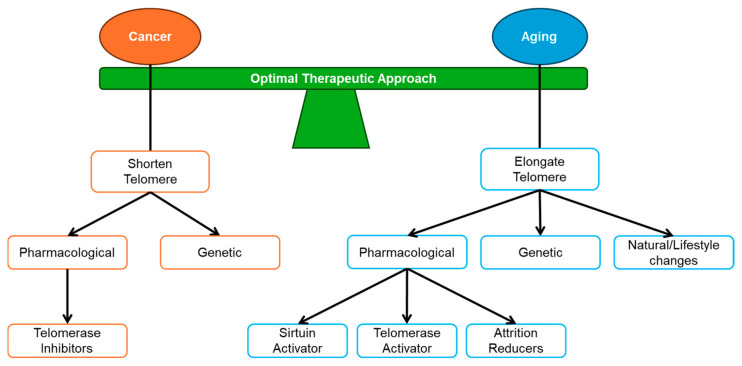
Optimal Therapeutic Approaches for cancer and aging. For better outcomes, there needs to be a balance between telomere elongation for aging and telomere shortening for cancer. For cancer, promising approaches include pharmacological and genetic approaches. For aging, promising approaches include pharmacological, genetic, and natural approaches.

The current literature has identified successful approaches, which, for example, avoid inducing senescence from cancer treatment. Apoptosis, another plausible outcome of telomere attrition that occurs when DNA damage reaches beyond a survivable threshold, is much more desirable. If this unrepaired DNA damage does not result in cell death, adverse outcomes such as genetic mutations and malignancy formation are more likely to occur [195]. Unfortunately, the cell type [196], genetic background [196], and the therapeutic dosages used on these cells all affect the result of such telomere shortening; thus, more information is required to determine the response by the cell to different damage [43]. These findings highlight the need for an Optimal Therapeutic Approach, which balances the induction of telomere erosion to treat cancer and the maintenance of telomere length to slow aging within normal somatic cells (Figure 7).

### 4.1. Optimal Pharmacological Approaches for Cancer

Some telomerase inhibitors, a class of chemotherapeutics, act on certain cancer types, sparing neighboring cells. Imetelstat (GRN163L) is a successful selective telomerase inhibitor that suppresses the assembly of telomerase [197,198,199,200]. The small, modified oligonucleotide targets the hTR region of telomerase, inhibiting telomerase function [39,200]. Due to most cancers relying on telomerase reactivation for proliferation, hTR inhibitors may have a selective effect on eliminating solely cancer cells. It is worth noting that past studies have shown Imetelstat to be associated with hematologic side effects. As per a 2018 study, the drug was able to selectively target myelofibrosis (MF) hematopoietic progenitor and stem cells, but only at lower doses since hematopoiesis was disrupted at higher dosages [201]. A previous study in 2015 found that Imetelstat was actively exerting its effect within MF patients yet also caused myelosuppression throughout treatment-related outcomes such as grade 4 thrombocytopenia in 18% or grade 4 neutropenia in 12% [202]. Following multiple clinical trials in recent years, Imetelstat was approved by the FDA to treat low- to intermediate-1-risk myelodysplastic syndromes (MDS) with transfusion-dependent anemia-resistant or ineligible for erythropoiesis-stimulating agents in June of 2024 [203,204]. MDS encompasses a group of cancers that affect the bone marrow, leading to the production of abnormal blood cells; about 30% of patients who received Imetelstat showed red blood cell transfusion dependence for ≥ 24 weeks, compared to the placebo group, which was about 3%. Like past studies, the most common side effects of Imetelstat include neutropenia and fluctuations in enzyme levels, such as alanine aminotransferase [203,204]. With FDA approval for only MDS, interest in the drug has sparked in its use beyond monotherapy due to the adverse side effects, which may be deemed unsuitable for some clinical applications. Imetelstat is currently undergoing multiple clinical trials as monotherapy, for more thorough testing, and in combination therapy for other cancers, such as acute myeloid leukemia (AML) and juvenile myelomonocytic leukemia (JMML) (see Supplementary File S4). Nevertheless, GRN163L presents a promising outcome in achieving an OTA following additional clinical testing to determine the acceptable dosages and conditions for where the drug may function without having a significant effect on nearby stem cells.

The current literature shows many other promising compounds in early preclinical phases, such as (1) BIBR1532, a telomerase inhibitor that impedes hTERT from interacting with hTR [205,206], and (2) Telomestatin, a telomerase inhibitor that stabilizes G-quadruplex (G4) structures [207,208,209]. These G-quadruplexes are secondary DNA structures that contain stacked G-tetrad planes of four guanine bases that are bound via hydrogen bonding [210]. They are capable of affecting telomerase binding to telomeric DNA and may prevent telomere elongation by the enzyme, thus having a beneficial impact on cancer therapy [211]. Currently, no clinical trials have been conducted using these two compounds based on ClinicalTrials.gov. Based on our review, there seem to be several papers showing potential for using balanced chemotherapeutics drugs to establish an Optimal Therapeutic Approach; however, clinical investigation is limited or absent for many of them (see Supplementary File S4).

### 4.2. Optimal Genetic Approaches for Cancer

Using gene therapy to target telomerase-associated genes is another approach used to treat cancer (see Table 1). CRISPR/Cas9 technology has been useful in specifically targeting the aberrant mutated pTERT, effectively reducing telomerase activity [212,213]. Selective control of telomerase inhibition remains an obstacle in gene therapy; however, oncolytic viruses may offer a promising solution. Oncolytic adenoviruses show significant specificity for cancer, where they can bind and enter cells expressing neoantigens not found in normal cells [214,215,216]. Upon attachment and entry, cancer-specific adenoviruses can replicate within the cancerous cells and suppress tumor growth up to 9 days [217] or 30 days [218] after transfection, with modified forms yielding even more powerful effects [219]. Furthermore, the use of antisense oligonucleotides (ASO), which vary in target sequence-specific regions of hTERT or hTR, yields merit from the ability to retain cell viability and telomere length while disengaging telomerase’s ability to promote cancer development [220,221,222]. ASOs reversibly downregulate the level of TERT expression within hepatocellular carcinoma [169] and cervical cancer [221], with the potential for additional specificity following conjugation using tumor-specific ligands [169].

While more research is needed for identifying and optimizing pharmacological approaches, drugs like Imetelstat show that the Optimal Therapeutic Approach is feasible in the realm of chemotherapy, where cancer is treated without significantly promoting aspects of aging such as senescence. Genetic approaches are still in the early stages of development, but we believe they hold great promise for more personalized therapies with less collateral damage compared to other treatments (see Table 1). Future research should take into consideration the impact of cancer treatment effects on aging or age-related complications, whether through direct impact on telomere length or through promoting contributors of aging.

## 5. Telomere Attrition in Aging

### 5.1. Contributors of Aging

#### 5.1.1. Senescence

**Figure 8:**
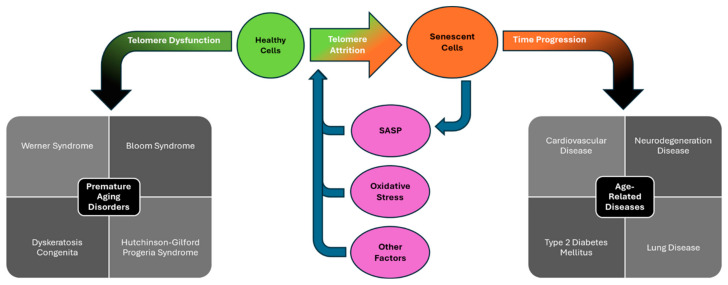
Effects of telomere dysfunction and telomere attrition. Telomere dysfunction is associated with the development of premature aging disorders such as Werner syndrome, Bloom syndrome, Hutchinson–Gilford Progeria Syndrome, and dyskeratosis congenita due to genetic defects (see Table 2). Telomere attrition can lead to senescent cells, which produce SASP, promoting telomeric damage. The accumulation of senescent cells can lead to age-related diseases, such as cardiovascular disease, neurodegeneration disease, lung disease, and type 2 diabetes mellitus. Figure colors are used for contrast only and do not represent any meaning beyond showing the different elements of the figure.

As people age, there is an accumulation of senescent cells [235,236,237] and a noticeable rise in the prevalence of chronic diseases [238,239]. Senescence is a state of irreversible cell cycle arrest [55,222,240] caused by several factors, including DNA damage, reactive oxygen species (ROS) [241], and especially telomere attrition [46] (Figure 8). According to the telomere hypothesis, senescence is triggered when the telomeres in somatic cells reach a critical length, hitting the Hayflick limit [49,242,243]. The loss of bases in the telomeric region damages the T loop and predisposes the exposed overhang to the DDR since the telomere is now recognized as a double-strand break (DSB), ultimately leading to senescence and apoptosis [21,46,244]. Additionally, senescence in immune cells promotes inflammation, which plays a role in advancing cardiovascular disease, diabetes, and other age-related diseases [245,246].

Senescent cells release common pro-inflammatory compounds, such as interleukin-6 and tumor necrosis factor alpha [59], into their local environment, which induces the senescence-associated secretory phenotype (SASP) [247]. A feature of senescence, SASP, occurs in parallel with irreversible cell cycle arrest, which prevents damaged cells from proliferating [64]. Despite the halted telomere attrition resulting from cell cycle arrest, the associated inflammation can contribute to the slow progression of aging-related diseases. This progression may occur through adverse effects on other cells within the microenvironment, such as excessive ROS induction [248], as well as senescent cell accumulation [59].

#### 5.1.2. Cellular and Mitochondrial ROS

The telomere–mitochondrial axis of biological aging integrates DNA damage and associated telomere attrition with p53 activation. As mentioned previously, p53 can lead to senescence as well as apoptosis if severe DNA damage occurs [196]. It may also disrupt mitochondrial function after suppression of PGC-1 alpha, a key metabolic regulator of the organelle and its downstream targets [249]. A feed-forward loop then occurs due to this organellar compromise—increased ROS elicits persistent DNA damage and DDR, which can lead to increased telomere attrition [241].

As one of the major contributors to aging, ROS can enhance inflammation [250] through activation of the AMPK pathway and redox-sensitive transcription factors, notably NF-kB [251] and nuclear factor activator protein-1 [252]. With the carbonyl group’s addition, oxidized macromolecules lose their functions, thus accumulating in cells that escape apoptosis and disrupt cellular functions [253,254]. Oxidative damage tends to cause telomere loss from the TTAGGG repeats in telomeres which exhibit heightened oxidative sensitivity [255]. On the organelle level, mitochondrial ROS is a significant factor in telomere-dependent senescence [256]. Mitochondrial dysfunction, which arises from the release of intracellular calcium from increased oxidative damage, leads to further ROS generation and telomere attrition that are both implicated in the progression of aging-related diseases [193,257].

### 5.2. Age-Related Diseases

Approximately 80% of adults that are older than 65 years old have at least one chronic disease, with 50% having the burden of multiple chronic diseases [258]. In many cases, senescent cells are present and cause structural degradation within healthy tissue, interference between adjacent cells, and the triggering of a dysfunctional immune response [259,260]. As mentioned, senescent cells release several damaging proteins, such as growth factors, protease, and inflammatory cytokines that affect normal cells [59,247], which have been linked to various diseases of aging, including cardiovascular and neurodegenerative diseases [56] (Figure 8 and Table 2).

#### 5.2.1. Cardiovascular Disease

Within the United States alone, the prevalence of cardiovascular disease has risen from ~40% in 40–59-year-olds to ~75% in 60–79-year-olds and 86% in those above 80 [261]. Cardiovascular disease (CVD), an overarching term that describes any ailment of the heart and the blood vessels, has remained the prominent cause of premature death globally [262]. Atherosclerosis occurs when damaged endothelial cells cause an inflammatory response, resulting in plaque lesions [263,264], with ROS and lipid peroxidation being two major causes of such plaque formation [265,266]. Due to marked declines in vascular system health with age, the plaques of aged individuals exhibit higher levels of senescent cells [267]. Shortened telomeres are associated with hypertension, coronary artery disease [268,269,270], vascular dementia [271,272], and myocardial infarction [273]. Both oxidative stress and chronic inflammation contribute to telomere dysfunction.

Mitochondrial dysfunction may cause higher oxidative stress, which in preclinical mouse studies has led to the thickening of the aorta [274,275]. Telomerase deficiency and telomere attrition impair cell division and promote cardiomyopathy and hypertrophy in mice [276]. Senescent vascular cells are only present in atherosclerotic lesions, expressing proinflammatory molecules and decreasing endothelial nitric oxide synthase, ultimately leading to atherosclerosis and vascular aging [277,278]. Furthermore, low levels of telomerase and short telomeres destabilize the atherosclerotic plaque, thereby increasing the risk of stroke and myocardial infarction [279].

Ultimately, replicative senescence influenced by ROS [256] and associated telomere attrition contribute heavily to vascular aging [280], which increases the risk of cardiovascular complications [256]. Past data from 2003 have shown that those with shorter telomeres in blood DNA have 3.18 times higher risks of mortality from heart disease, and patients with coronary artery disease and chronic heart failure tend to have significantly shorter telomeres [281]. Such connections are also seen in immune cells, where patients with premature myocardial infarction had shorter leukocyte telomeres [273]. The 2003 case–control study examined 203 cases that had a premature myocardial infarction and 180 controls. Researchers reported a three-fold increased risk of premature myocardial infarction before reaching 50 years of age within individuals exhibiting telomere lengths shorter than average following mean terminal restriction fragment (TRF) length examination in leukocyte DNA [273]. These findings suggest that telomere attrition plays a significant role in cardiovascular aging and disease, though the strength of the relationship between telomere length and specific cardiovascular conditions varies.

#### 5.2.2. Type II Diabetes Mellitus

It is predicted that the number of individuals affected by Type II diabetes mellitus (T2DM) will increase by more than fourfold from 2005 to 2050 [282]. With the increased prevalence of T2DM globally, researchers have examined telomere biology as a possible insight into the pathology of the disease. Some studies have found that telomere length attrition is not associated with whether the patient has diabetes [283]. For example, a 2024 molecular epidemiological study found a substantial inverse correlation between increasing age and the telomere length of 121 diabetic individuals. When a patient is diagnosed with obesity, such as class 3, shorter median telomere length within PBMCs is associated with higher BMI [283]. In contrast, it was also observed that non-obese patients versus those with pre-obesity have a longer median telomere length but with lower BMI [283]. A 2013 meta-analysis investigating the association between T2DM and telomere attrition found that the connection remains controversial because of conflicting results potentially from small sample sizes in some studies [284]. Nonetheless, preclinical data conducted on embryonic stem cells with hyper-long telomeres within mice have shown less LDL, as well as increased insulin and glucose sensitivity [285]. Additionally, it has been shown that *Terc* -/- G4 mice have an impaired replicative capacity of pancreatic beta cells, resulting in lessened islet size [286]. This impairment impedes insulin release and causes glucose intolerance, thereby enlisting diabetes mellitus as an age-related disease [286]. These findings suggest that telomere length plays a role in metabolic regulation, even though the relationship between T2DM and telomere attrition is inconsistent, calling for further research into telomeric elements as therapeutic targets for T2DM management.

#### 5.2.3. Neurodegenerative Disease

The two hallmarks of Alzheimer’s disease (AD) are aggregated beta-amyloid peptide and intraneural accumulation of neurofibrillary tangles of tau, which are misfolded and hyperphosphorylated [287,288,289]. As per the findings of a 2014 review on SASP-related drivers of aging pathologies, beta-amyloid peptides were shown to trigger senescence and massive production of IL-6, an abundant cytokine within the CNS of AD patients [267]. In addition, activated macrophagic microglia cells surround the amyloid plaques in the brains of those with AD and become pro-inflammatory, thereby damaging neurons and synaptic connections [267].

Telomere length has the potential to predict deaths among patients that experience AD, dementia [290], or stroke [291], with shortened telomeres identified as a risk factor for AD following analysis within PBMCs [290,292]. Shortened telomeres are associated with vascular dementia [271,272] and worsening cognition in women affected by dementia [293]. Telomere attrition is associated with increased multiple sclerosis symptoms, with shorter leukocyte telomere length associated with increased disability and decreased brain volume [294]. In fact, for relapsing multiple sclerosis patients, a 2024 article mentions that their telomere lengths are associated with the time in which multiple sclerosis progresses within 10 years [295]. Preclinical studies show that when mice are prone to senescence, they experience neuronal degeneration that leads to the atrophy of the olfactory bulb and forebrain, leading to age-related learning and memory deficiencies [274,275]. These findings suggest that telomere length is linked to neurodegenerative processes, particularly in diseases like AD and multiple sclerosis. Although the exact relationship between telomere attrition and these conditions remains under investigation, the evidence points to telomeres as potential biomarkers for disease progression and therapeutic targets for neuroprotection, warranting further research.

#### 5.2.4. Lung Disease

Cigarette smoke has been labeled as the most preventable source of morbidity and mortality in the United States [296]. Cigarette smokers are 15 times more likely to develop lung cancer and 11 times more likely to develop chronic lung disease compared to those who do not smoke [297]. Cigarette smoke provokes an inflammatory response from increased oxidative stress placed on fibroblasts, alveolar cells, and epithelial cells [298,299]. Such stress places a burden on the cells and promotes telomere attrition, which causes dysregulation of several cellular functions. Structural cells within the alveolar wall [300] begin to undergo apoptosis along with degradation of the lung tissue, thus leading to chronic bronchitis [301] and emphysema [302]. Cigarette-smoking-induced oxidative stress also promotes SIPS from the direct activation of p53, p16INK4a, and later p21 [299,300]. PGC-1 alpha is also upregulated, which results in disturbed mitochondrial biogenesis, leading to increased ROS and lessened autophagy in airway epithelial cells [303]. Researchers have demonstrated that senescence-prone mice had senile lungs linked with higher oxidative stress, partially from mitochondrial dysfunction [274,275]. Upregulated levels of p21 and p53 have been linked with club cell senescence in patients with Chronic Obstructive Pulmonary Disease (COPD), a condition characterized by the fixed or partially reversible disruption of airflow within the lungs [304,305] that impairs normal regeneration of airways and promotes harmful, excessive inflammation [303].

The association between the occurrence of replicative senescence and premature lung aging has also been studied extensively with the pathogenesis of COPD. Telomere attrition impacts the normal functionality of lung tissue, restricting the replicative ability of the aforementioned club cells (formerly known as Clara cells), which are progenitors of the peripheral airway epithelium [304,306]. These cells are involved in tissue repair and have a high expression of senescence markers in COPD patients [304]. Critically short telomeres activate DDR proteins such as ataxia telangiectasia mutated (ATM) as well as downstream effectors such as p38 mitogen-activated protein kinases (MAPKs) [307]. MAPKs are involved in cell signaling as well as control of inflammatory processes [308,309]; thus, their increased activation amplifies SASP, thereby degrading lung tissue over time [304] and inducing airway remodeling [310]. Together, oxidative stress and telomere attrition show a strong connection to the pathogenesis of lung diseases such as COPD, which highlights the importance of understanding the effects of telomere attrition on lung tissue and finding more approaches to reduce the associated cascade of undesired events.

### 5.3. Telomere and Progeroid Syndromes

Telomere syndromes are rare diseases that involve short or dysfunctional telomeres; therefore, they are associated with shortened lifespan and early death due to accelerated biological aging (Figure 8 and Table 2) [111,311]. One premature aging disorder has been labeled as a telomere syndrome as well—dyskeratosis congenita [311]. The condition is caused by mutations within proteins that are associated with telomerase, such as those that affect the hTERC stabilizer dyskerin [312,313,314]. On the other hand, progeroid syndromes, such as Werner [315] and Bloom [316,317,318], involve inherited DNA repair defects that increase DNA damage and accelerate biological aging [319]. Almost all progeroid syndromes are associated with short telomeres, and those such as Werner and Bloom occur independent of telomerase [320,321]. Progeroid syndromes often involve similar symptoms, including immunodeficiency and cancer [322]; however, it should be mentioned that not all progeroid syndromes involve accelerated telomere shortening [323,324].

**Table 2:** Age-related diseases and premature aging disorders.

Condition Name	Relation to Telomere Attrition	Source
Atherosclerosis	Plaque caused by endothelial cells’ inflammation	[263,264]
Type 2 Diabetes Mellitus	Insulin resistance, with early onset related to short telomeres	[283,285,286]
Alzheimer’s Disease	Neurodegeneration associated with beta-amyloid and phosphorylated tau, which connects to senescence	[325,326,327]
Chronic Obstructive Pulmonary Disease (COPD)	Airflow disruption in the lungs, which connects to senescence and telomere attrition	[304,307,310]
Pulmonary Fibrosis	Scarring of the lungs caused by telomere loss and mutated telomerase	[328,329]
Liver Fibrosis/Cirrhosis	Scarring of liver, which has a connection to telomere attrition and altered telomerase	[169,330]
Osteoporosis	Weakened bones, which have a connection to telomere attrition or telomerase mutation	[331,332]
Parkinson’s Disease	Neurodegeneration from alpha-syn aggregation, whose development is accelerated by senescence	[333,334]
Werner Syndrome	Mutation in *WRN*, which works with TRF2 to activate helicase	[315,316,335]
Bloom Syndrome	Mutation in *BLM*, which works with TRF2 to activate helicase	[316,317,318]
Dyskeratosis Congenita	Mutation in Dyskerin, which ensures TERC’s proper structure	[312,313,314]
Hutchinson–Gilford Progeria Syndrome	Mutation in *Lamin A* that impairs telomere	[336,337,338]
Nijmegen Breakage Syndrome	Mutation in *NBS1*, which helps repair TRF2	[339,340]
Cockayne Syndrome	Neurodegeneration, mainly caused by mutation of the *CSB* gene, which encodes the DNA repair protein. CSB maintains telomere length upon interaction with TRF1 and TRF2	[341]

Several age-related diseases (in blue) and premature aging disorders (in orange) associated with telomere attrition were identified in the PubMed database. Refer to Supplementary File S2 for the complete table.

#### 5.3.1. Werner and Bloom Syndrome

Werner syndrome is an autosomal recessive condition caused by a mutation to the gene *WRN* [316,335,342], which codes for RecQL2 [343]. The WRN-helicase complex is one of the major enzymes within the RecQ family responsible for ATP-dependent DNA unwinding and 3′–5′ exonuclease activity, which is responsible for DNA replication and repair [344,345,346]. Afterward, TRF2 recruits WRN to remove nucleotides forming pathological 3′ overhang substrates on telomeres [347] while also activating the WRN protein’s helicase activity [343]. Fibroblasts of such patients would exhibit accelerated telomere attrition and premature senescence [348]. Moreover, individual sister chromatids are observed to experience telomere loss, which ends in chromosome breakage–fusion events, which further destabilize the genome and accelerate senescence [349]. The *WRN* mutation causes maintenance dysfunction of the telomeric region, accelerating replicative senescence and genomic instability, which may contribute to premature aging and potentially cancer [350].

Bloom syndrome is caused by a mutation to the gene *BLM*, which codes for another RecQ helicase [317,318]. TRF2 activates BLM protein to stimulate its helicase activity, which is involved in DNA replication and repair [316]. Thus, like Werner syndrome, the telomere length is also not directly reduced, but the rate at which telomere attrition occurs is accelerated by the defects in the machinery involved in DNA replication and repair [351]. Such acceleration contributes to genomic instability, immunodeficiency, and growth retardation [320,352,353].

#### 5.3.2. Dyskeratosis Congenita

Dyskeratosis congenita is caused by an X-linked recessive or an autosomal dominant mutation and involves the alteration of dyskerin [312,313,314]. Dyskerin is a nucleolar protein coded by X-chromosome’s *DKC1*, which helps modify specific small RNA molecules, especially ribosomal RNAs and TERC [172,354]. Specifically, dyskerin is required for proper telomerase RNA folding and stability [172,312]. In the X-linked recessive form, dyskerin becomes mutated, reducing TERC production and, in turn, reducing telomerase activity and telomere length [355]. The autosomal dominant form commonly involves TERC and TINF2 mutations; therefore, this form is associated with telomerase [356] or mutations in shelterin proteins [56,357].

Dyskeratosis congenita is progressive and presents with signs of abnormal pigmentation leukoplakia [358]. Death at a young age is also a common adverse outcome associated with the disrupted regeneration of the cells within the affected bone marrow issues. Telomere shortening is substantially accelerated within cases of inherited bone marrow failure syndrome (BMFS), especially dyskeratosis congenita [359]. This is due to the high turnover rate, thus lessening telomere length over time of hematopoietic cells, which include the proliferative stem cell population [360]. That said, dyskeratosis congenita is a condition that involves accelerated telomere attrition in hematopoietic cells and clinical hematopoietic failure [356], leading to progressive bone marrow failure [154] and premature death [314,361]. In fact, developing bone marrow failure occurs in more than 80% of cases [355].

#### 5.3.3. Hutchinson–Gilford Progeria Syndrome

Hutchinson–Gilford Progeria syndrome (HGPS) is an autosomal dominant illness that is estimated to be present in 1 in roughly 4 million live births [347]. HGPS is an accelerated aging syndrome that involves genetic mutation for the nuclear lamina’s proteins, specifically caused by point mutations in *Lamin A* [336,337,338]. When mutated, LMNA prevents the DNA damage response toward telomeres, which may accelerate cell senescence [362,363]. Children with HGPS show telomere lengths resembling those of 70-year-old patients [248,364]. Furthermore, such alterations in the nuclear lamina compromise not only the nuclear structure but also the condensation of genetic material into heterochromatin [365] and the components involved in DNA replication and repair [366]. These events all have the potential to induce telomere attrition from the resulting nuclear instability [363]. Clinical presentations of premature aging disorders include short stature, lipodystrophy, osteoporosis, and a higher risk of strokes [364,367]. Strikingly, the progeroid condition generally results in death at approximately 13 years of age from either stroke or myocardial infarction [368].

## 6. Optimal Therapeutic Approaches for Aging-Related Diseases and Premature Aging Disorders

While there are no current treatments to cure premature aging disorders and age-related diseases, many promising compounds can mitigate the symptoms and halt progression. However, like cancer, a balance is needed to minimize systemic damage and not increase the risk of developing further complications. For example, the maintenance of corneal clarity in vivo depends on the density of corneal endothelial cells, which maintains the endothelial pumping function [369]. In such a situation, the use of senolytic drugs will not only destroy senescent cells but also lower the density of cells, leading to visual clarity issues [369]. Therefore, it is better to revert the senescent phenotype using resveralogue or similar compounds rather than a senolytic [369]. These findings highlight the necessity for the Optimal Therapeutic Approach in aging (see Table 3).

### 6.1. Optimal Pharmacological Approaches for Age-Related Diseases and Premature Aging Disorders

#### 6.1.1. Sirtuin Activators

Sirtuins, mammalian protein deacetylases, have up to seven forms within humans (SIRT1-7), with SIRT1 being the one suggested to expand lifespan and quality of life [370,371]. SIRT1 acts via deacetylation and stimulation of PGC-1 alpha [372,373]. SIRT1 interacts with telomeres [374] and maintains telomere length via modulation of telomere shelterin TPP1 in mesenchymal stem cells [375]. The protein is also influential in regulating FOXO3 [376], a stress-response transcription factor, which can act as a tumor suppressor in certain cancers, halting aerobic glycolysis in tumors [377,378]. FOXO-3 is also known to modulate a diverse range of biological functions, including but not limited to managing cell cycle checkpoints and arrests, ROS detoxification, damaged DNA repair, and apoptosis [379]. This homeostatic maintenance, induced by the regulation of the FOXO-3 protein, highlights the importance of SIRT1 for aging and cancer [106]. One of the most extensively studied compounds that aid in reducing the effects of aging is the anti-oxidant and anti-inflammatory known as resveratrol, a polyphenolic compound [380,381,382,383]. Resveratrol is a potent SIRT1 activator that is effective in smaller animals [384,385], where SIRT1 has been shown to prevent telomere attrition via telomerase regulation [386]. Resveratrol improves mitochondrial function [387,388] and prevents metabolic diseases via PGC1 alpha activation alongside SIRT1 [389]. Analogs of resveratrol as well, specifically SRT1720, which is the most potent of them all, have been 800–1000-fold more effective than resveratrol in activating SIRT1 while also protecting against lung inflammaging and reducing lung cell senescence [389]. Due to these findings, there has been an increased interest in examining resveratrol in different conditions, such as cancer therapy. A recent clinical trial that concluded in 2022 found that resveratrol in combination with sirolimus has led to a modest improvement in patients with lymphangioleiomyomatosis, a rare systemic metastasizing neoplasm in the lungs [390,391]. With constant inflammatory status deemed a hallmark of aging, the term “inflammaging” refers to the enhanced systemic inflammation that is both chronic and consistent and is closely linked with the development of aging-related diseases [392].

A mammalian SIRT1 activator similar to resveratrol, known as quercetin, protects against the development of emphysema [393,394] and ovarian aging due to anti-oxidant effects [395]. Polyphenols similar to quercetin, such as Butein, Piceatannol, and Myrcetin, are all able to activate SIRT1 as well but 3.19 to 8.53-fold higher than that of resveratrol [376], thereby having more potency on reducing age-related effects. Fucoidan is another beneficial sirtuin activator, having been shown to increase the expression of SIRT6 almost 14-fold [396]. This SIRT6 upregulation improved cognitive impairment in mouse models that were induced by lipopolysaccharides (LPS), which are the pro-inflammatory components of Gram-negative bacteria cell walls and serve as models for molecular assessment for acute inflammation [397]. While the literature shows great potential for resveratrol analogs, such as SRT1720, and all these SIRT1 activators, according to Clincialtrails.gov, there are very few clinical studies being conducted on them (see Supplementary File S4).

#### 6.1.2. Telomerase Activators

The controlled activation of telomerase is another strategy that can slow age-related telomere attrition. AGS-499 is an example of a telomerase activator that protects cultured hippocampal neurons against amyloid-beta-induced cytotoxicity [398] and can also delay the progression of amyotrophic lateral sclerosis within SOD1 transgenic mice [399]. TA-65 (Cycloastragenol), a TERT gene activator [248], can increase the length of short telomeres within mouse embryonic fibroblasts haploinsufficient for the RNA component of telomerase in vitro [400]. The same study also showed that an adjusted dose of TA-65, administered daily for four months within female mice aged one or two years old, led to a 10-fold increase in telomerase RNA levels within the liver of the treated mice versus untreated mice of the same age; no significant increase in carcinogenesis following prolonged supplementation between the same-age treated and untreated groups was observed [400]. A randomized, double-blind, controlled study conducted across one year with 117 healthy, cytomegalovirus-positive individuals from age 53 to 77 showed statistically significant telomere lengthening after the use of low-dose TA-65, though the incidence of cancer was not examined [401]. As far as we know, no clinical trials are investigating AGS-499; on the other hand, there have been many completed and active trials on TA-65 concerning age-related diseases. A 2018 mouse model study demonstrated that TA-65 allows for T-lymphocyte proliferation in a telomerase-dependent manner [191]. A 2023 study on CVD patients with a history of myocardial infarction mentioned that TA-65’s main mechanism of action is yet to be confirmed due to unaffected nuclear telomerase activity, thus potentially non-telomeric effects, at the trial’s conclusion [402]. An active clinical trial seeks to test TA-65’s possible improvement of microvascular dysfunction, and it is estimated to end in 2029 (NCT05598359) (see Supplementary File S4) [403,404]. Statins, like atorvastatin, are proven CVD prevention strategies that have been shown to elicit telomerase activation [405,406]. Specifically, they stop the loss of the TRF-2 protein within endothelial progenitor cells [407], halting telomere attrition while still promoting DNA repair [406,408]. For premature aging disorders, there is a peptide compound called GSE24.2 that is derived from a dyskerin peptide domain known to interact with hTR [409]. This peptide was then designed to treat dyskeratosis congenita by stopping the inhibition of telomerase and increasing TERC and TERT expression, thereby increasing telomerase activity [189,409].

#### 6.1.3. Reducers of Telomere Attrition

Another strategy is to limit the telomere attrition rate. Smoking is a destructive habit associated with high states of oxidative stress, causing substantial increases in telomere erosion [219,410]. The non-hydrolyzed forms of carnosine and carcinine can reduce telomere attrition rates and telomeric DNA damage in COPD patients via telomerase activity modulation and redox regulation [411,412]. There are no active or completed clinical trials for carcinine; however, several exist for carnosine, including one estimated to finish in 2024. The phase 1/2 trial examines peripheral arterial disease, a disease caused by occlusion due to atherosclerosis, one of the main age-related diseases [413]. Additionally, eliminating harmful chemicals from a person’s system can slow aging processes.

Melatonin has been found to combat ROS by regulating telomeres and mitochondrial functions, thus controlling vascular aging and decreasing atherosclerosis age-related endothelial injuries [98]. It also has been found to delay ovarian aging within the oocytes of mice [414]. Other antioxidants, such as coenzyme Q10, have a similar regulation of mitochondria and ROS [415], and N-acetyl-L-cysteine (NAC) has been found to restore ROS-induced telomere attrition in vitro in mouse oocytes [416] by quenching ROS [193]. For those affected by diabetes, glycemic control induced via careful sugar intake in everyday food consumption can reduce additional telomere attrition [417].

**Table 3:** Promising pharmacological approaches for aging.

Name	Mechanism of Action as Described in Source	Source
Melatonin (Antioxidant)	Regulates ROS levels and telomeres, affecting vascular aging; decreases atherosclerosis’s age-related endothelial injuries; delays ovarian aging in oocytes.	[98,414]
N-acetyl-L-cysteine (NAC, Antioxidant)	Helps restore ROS-induced telomere attrition within oocytes in vitro; suppresses ROS and increases glutathione.	[193,416,418]
Metformin	Mitigates age-related metabolic changes, activates AMPK, and boosts SIRT1 activity.	[419,420,421,422]
Danazol	Promotes telomere elongation and suppresses attrition in individuals diagnosed with premature aging disorders.	[423]
TA-65 (Cycloastragenol)	An activator of the TERT gene without increasing incidence of cancer within animal models; T-lymphocyte proliferation in a telomerase-dependent manner in mice.	[191,400]
Statins	Halts telomere attrition by stopping loss of TRF2 in endothelial progenitor cells; proven CVD prevention strategy that works via telomerase activation.	[405,406]
Resveratrol	A potent SIRT1 and PGC-1 alpha activator, which has been shown to prevent telomere shortening via telomerase regulation and mitochondrial function.	[384,388,394,424,425,426]
Rapamycin	A mTOR complex 1 inhibitor that lengths lifespan within mice, worms, and flies.	[427,428,429]
Curcumin	Activates SIRT1 and AMPK; inhibits mTORC1 and tau aggregation, protecting against diabetes, CVD, and Alzheimer’s.	[430,431,432]
GSE24.2	A peptide that can help dyskeratosis congenita by recovering telomerase activity and raising antioxidant levels.	[189,409,433]

An overview of existing and novel drugs with potential in managing age-related diseases (in blue) and premature aging disorders (in orange). Refer to Supplementary File S5 for the complete table.

### 6.2. Optimal Genetic Approaches for Age-Related Diseases and Premature Aging Disorders

Telomerase gene therapy delivers TERT effectively into a gene and has been shown to slow cell aging and increase lifespan without increasing tumorigenesis within mice [230,233]. AAV-9-based gene therapy, specifically in mice models, has allowed for telomerase activation via adenoviral transfection, which has directly reduced incidences of osteoporosis; sugar intolerance; and neuromuscular dysfunction, pulmonary fibrosis, and myocardial infarction-induced necrosis of heart tissue [230,434]. The ectopic expression of TERT via AAV-9 also did not lead to an earlier onset of cancer, thereby supporting the notion that ectopic expression of the genes that regulate telomeric maintenance does not necessarily induce cancer development [435]. While more research is needed to validate the efficacy of such genetic approaches in individuals, preclinical studies show great promise.

With respect to premature aging disorders, a 2019 study using a transgenic HGPS mouse model revealed that progerin-induced telomere dysfunction leads to the transcription of telomeric non-coding RNAs (tncRNAs) [436]. These tncRNAs helped to improve tissue homeostasis, and they increased the median lifespan by 24% when compared to control ASO-treated mice [436]. Telomere dysfunction induces the transcription of specific RNAs, such as telomeric damage-induced long noncoding RNAs and telomeric DNA damage response RNAs [437,438]. These RNA transcripts are crucial for DDR activation and maintenance; therefore, telomeric sequence-specific antisense oligonucleotides (tASOs) can inhibit these RNA transcripts and may then prevent activation of the DDR and premature cellular senescence [436]. For Werner syndrome, a team of researchers reported reprogramming three fibroblast cell lines derived from patients to induced pluripotent stem cells (iPSCs), which resulted in telomere elongation and telomere dysfunction prevention [439]. Initially, the researchers attempted to reprogram the cells using the Sendai virus encoding the Yamanaka factors (Oct-4, Sox2, Klf4, C-Myc); however, the induction rates were low, and some plates contained no iPSCs [439]. These fibroblasts showed negligible levels of telomerase activity, and upon transfecting hTERT via a plasmid, the team observed better survival and an increased number of iPSC compared to cells without ectopic expression of hTERT [439]. This approach serves as an alternative method to produce iPSC efficiently. iPSC holds great potential in studying disease pathology; moreover, they can differentiate into three germ layer cells and mesenchymal stem cells (MSCs) [439,440]. These MSCs displayed a greater rate of proliferation and decreased senescence despite the mutated WRN protein. As far as we know, such genetic approaches remain in the preclinical phase; nonetheless, they hold the potential to deliver an OTA for premature aging disorders.

### 6.3. Natural Approaches and Lifestyle Changes for Better Aging Outcomes

#### 6.3.1. Dietary Diversification

The data show that diversified diets promote healthier aging. Diets such as the Mediterranean or Okinawan are rich in vitamins A, E, and C, as well as minerals and phytochemicals, which are shown to activate Nrf2 and sirtuin proteins [441,442]. In fact, the Mediterranean diet specifically is associated with activated telomerase expression, thereby regulating telomere maintenance [443]. Also, the diet may decrease the risk of developing Alzheimer’s or Parkinson’s disease [444,445].

Omega-3 intake is associated with a lower risk of premature death from CVD, breast cancer, and other conditions [446,447]. A recent 2024 study showed that omega-3 fatty acids had a positive effect on leukocyte telomere length by demonstrating a significant difference in length between mice treated with omega-3 fatty acids and mice that were not [448]. Cognitive improvement via neuronal growth, memory formation, and general nerve maintenance alongside linked improvement in glucose and lipid disorders through PGC1-alpha transcription regulation [449] are additional benefits when omega-3 is incorporated within the diet [450,451].

Vitamin B3, or nicotinamide riboside, can prevent telomere-induced DDR and works against inflammatory processes within humans, which helps restore a healthier microenvironment and does not promote telomere attrition [452,453]. Folic acid supplementation displays an ability to reduce intracellular ROS and homocysteine [454], which is considered to be an indicator of accelerated telomere attrition and endothelial cell senescence [455].

#### 6.3.2. Dietary Restriction

Behavioral changes such as the implementation of caloric restrictions (CR) have been deemed one of the most examined interventions shown to improve the health span of many organisms, including yeast (*Saccharomyces cerevisiae*) [456], worms (*Caenorhabditis elegans*) [457], flies (*Drosophila*) [458], mice [459], and even humans [460] through extensive and widespread investigation [461,462,463]. CR refers to lessened nutritional intake that does not cause malnutrition [464]. Within mice, the intervention was shown to reduce mutation frequency, thereby lowering the accumulation of endogenous genotoxic products formed from altered metabolic processes invoked by the caloric deficit [465]. With regard to the underlying pathways by which CR works, the extended lifespan of *S. cerevisiae* [466] and *Drosophila* [467] has been shown to occur via Sir2 mediation. The attenuation of stress-induced apoptosis within tissues from 12-month-old rats that underwent CR was caused by SIRT1 induction [468]. Deacetylation of the DNA repair factor Ku70 inhibited the release of the pro-apoptotic Bax factor, thus preventing the removal of irreplaceable cells and extending lifespan [468]. Also, the expression of SIRT1 was approximately two times higher in HEK 293T cells cultured with CR serum, which was obtained from the rat model when compared to those cultured with ad libitum serum [468]. Reduced glucose intake is sensed by AMP-activated protein kinase, which has been associated with decreased mTOR signaling [469]. Across the various model animals used to study the longevity effects of this intervention, decreased insulin/IGF signaling and reduced activation of mTOR are two of the most documented outcomes associated with lifespan extension by CR [470,471,472]. Aside from short-lived species, rhesus monkeys have also been studied for the effects of CR on lifespan. For example, rhesus macaques that followed a 30% CR plan had roughly half the mortality rate of the monkeys fed as desired [473]. For rhesus monkeys in captivity, the median reported survival age was approximately 26 years, and the maximum lifespan reached about 40 years [474]. A study conducted by the National Institute of Aging reported that one-third of the monkeys placed under late-onset CR lived for more than 40 years [473,475]. With regard to human studies, it should be noted that some of the caloric restrictions used are not practical for a person to follow, demonstrating a great limitation of this lifestyle change in the study of CR on humans.

#### 6.3.3. Physical Activity and Stress Management

Physical exercise has also elucidated epigenetic effects on the prevalence of telomere attrition and neurodegenerative disease. Physical activity has been positively associated with longer telomeres within PBMCs in low-risk prostate cancer patients [476] as well as normal, healthy twin volunteers from the general UK population [477]. A recent 2022 study emphasizes that physical activity can increase the POT 1 expression, the protein that encodes for the protective capping proteins on telomeres, thereby promoting beneficial telomere length maintenance within the blood cells of horses [478]. Following evidence-based approaches to combat CVD, it has been shown that exercise alongside statin compounds has highlighted activation of telomerase activity [479]. Both single or continuous forms of exercise, such as endurance training, resulted in increased telomerase activity and telomere length within human PBMCs [480]. Increased expression of TERT and heightened telomerase activity have also been observed within the PBMCs, non-cancerous somatic cells, and epithelial tissue of rodents [481].

Under the umbrella term known as ‘mindfulness-based stress reduction’ (MBSR), practices such as yoga and meditation [482] are techniques known for their beneficial mental effects, such as mediation of stress, anxiety, and depression [483,484,485]. These psychological treatments have also been shown to have physical impacts on health, where an increase in psychological stress has been linked with reduced telomerase activity and telomere shortening [476,486]. Individuals who harbor mood disorders are also known to have shorter telomeres compared to their healthy counterparts [487]. Diminishment of psychological distress positively impacts telomere maintenance, with stress management being shown to result in heightened telomerase activity in peripheral mononuclear cells after a three-month timeline [257,488]. A cross-sectional study comparing long-term mindfulness meditators (10 years) and a control group also showed a significant reduction in telomere erosion and DNA methylation in specific genes [489]. All these findings support the notion that combining a diversified diet with an active lifestyle and mindfulness practices delivers the greatest benefits and better aging outcomes for patients and healthy individuals.

## 7. Optimal Dual-Action Approaches

### 7.1. Rapamycin

There are promising therapeutic approaches that can be used for cancer and aging, such as rapamycin, a mTORC1 inhibitor. Rapamycin, known to be inherently immunosuppressive [490], can inhibit the growth of some cancers; it affects breast cancer cell lines by slowing down the rate of cell proliferation and cell cycle progression at the G1 phase without affecting telomeres and telomerase activity [491]. It has also displayed the ability to extend life within flies [471] and C. elegans worms [492] by inhibiting the mTORC1 pathway. With regard to other preclinical studies, rapamycin administered to male and female mice at 600 days of age showed median and maximal lifespan extension by 9% and 14%, respectively [493]. Also, a 2016 study showed that 3-month rapamycin treatment was capable of increasing median life expectancy by up to 60% within 20-/21-month-old C57BL/6 mice, and it was emphasized that the rapamycin need not be administered continuously but rather at minimal dosages for such a benefit [494]. Based on several well-executed studies in mice and flies, rapamycin treatment seems most effective when administered early in life or even at birth [495,496]. A 2022 study reported that early-onset rapamycin treatment immediately significantly increased the lifespan of *Mus musculus* (house mouse) while using the same treatment later in life had no impact on lifespan [497]. Similarly, in UMHET3 male mice, rapamycin treatment from birth to day 45 increased the median lifespan by 11.8% [496]. While such duration seems brief, mice have shorter lifespans than humans. Thus, the equivalent for humans might be several years of rapamycin usage, underscoring the delayed effects of rapamycin on aging.

Additionally, the 2022 study reported that, by day 30, rapamycin-treated mice were three times smaller than control mice, which highlights another major concern [497]. Such negative growth outcome is also seen in children with renal transplants, where rapamycin treatment for 12 months significantly decelerated growth [498]. Therefore, rapamycin might be a better option for post-growth adults. Interestingly, in older male mice, continuous rapamycin treatment beginning at 20 months extended lifespan as effectively as when started at 9 months [427,493]. Remarkably, a 90-day high-dose treatment at 20 months increased the health span by 60% within middle-aged mice [494]. These findings suggest that late-life treatments require higher doses to achieve effects comparable to early-life regimens. However, early- or late-onset treatment with rapamycin in mice [497] and even lifelong treatment within *Drosophila* [495,497] seem safe as they did not elicit increased mortality. Finally, according to the literature, rapamycin decelerates the development of age-related diseases but does not treat terminal stages of diseases associated with loss of function [499]. In summary, rapamycin might act as an age-specific treatment that mitigates aging and age-related diseases when used at the right dose and timing, ideally as a preventative measure once growth is complete.

A 2020 clinical trial (PEARL, see Supplementary File S4) examined the weekly intake of 5 mg and 10 mg doses of rapamycin in healthy aging adults compared to a placebo [500,501]. The study enrolled more than 100 participants and measured several factors, including changes in bone density, blood markers, insulin, and liver functions after 6 and 12 months of the intervention [501]. In 2024, the Phase II study was updated and demonstrated two important findings when analyzing health span in older adults: safety and efficacy. The side effects were similar for all groups, the two experimental groups and the control group, showing that overall, rapamycin is relatively safe even after weekly chronic use for 12 months [501]. Additionally, there were no noticeable alterations in blood markers and visceral fat between all treatment groups [501]. Regarding the efficacy of the 10 mg dose, the men experienced an enhancement in bone density, while the women participants had improved lean body mass and a decrease in self-reported pain [501]. Findings show tolerability of long-term use of rapamycin and modest improvement, which are possibly sex-linked; nonetheless, it should be noted that the results are not yet peer-reviewed. The potential for rapamycin in clinical settings for cancer remains under exploration with respect to prevention (e.g., NCT04375813) and treatment of cancer (e.g., NCT02975882) (see Supplementary File S4).

### 7.2. Berberine

Berberine is a natural neuroprotective compound [502,503] shown to extend the life of various cell lines and mice [370]. Also, it is a chemosensitizer for cancer using AMPK activation [504], which downstream regulates SIRT1 and mTOR suppression to trigger autophagy [505]. As mentioned, SIRT1 interacts with telomeres and maintains telomere length via modulation of telomere shelterin TPP1 in mesenchymal stem cells [506], making berberine a promising telomere-protective drug with practical bioactivity in cancer and age-related diseases. A phase 2/3 clinical trial examined the connection between 500 mg twice a day of berberine and the prevention of CVD in 84 male participants, as previous studies showed possible sex-based benefits [507,508]. The researchers measured BMI, lipid levels (including LDL, HDL, and triglycerides), and blood pressure after 8 and 12 weeks [507,508]. The data were compared to baseline values and participants who took a placebo for the same duration [507,508]. The researchers reported no statistically significant changes in triglycerides and blood pressure between the two groups; however, male participants experienced an increase in testosterone and a decrease in LDL [507,508]. Regarding cancer, a clinical trial tested the safety and efficacy of berberine in preventing colorectal cancer that included almost a thousand participants who had previously had the cancer [509]. The researchers had 553 participants take 300 mg tablets twice a day for 3 years while the control group took a placebo, measuring the incidence rate of colorectal adenomas (a precancerous lesion) and their size if present [509,510]. After 3 years of trial duration, the intervention yielded statistically significant recurrences of adenoma upon follow-up: 36% of the berberine group and 47% of the placebo group. Neither group developed colorectal cancer [509,510]. The preclinical and clinical data show the safety and efficacy of berberine, but more research is needed to optimize the dose and uncover other therapeutic benefits.

### 7.3. Other Dual-Action Drugs

Alongside its neuroprotective activity [511,512], the telomerase activator and astragaloside AG-IV has been gaining popularity in its capacity to combat a variety of degenerative diseases, such as age-related macular degeneration [513] or metabolic syndrome [514]. The compound exhibits attributes ideal to the Optimal Therapeutic Approach; AG-IV has been shown to be anti-inflammatory via interleukin-1beta inhibition when treating rats with induced rheumatoid arthritis [515]. AG-IV has also been effective in improving glucose tolerance as well as endothelium-dependent vasorelaxation of the aorta within fructose-fed rats [514]. Anti-cancer activity in vivo has been demonstrated through the restriction of tumor growth within lung carcinoma cells of C57BL/6 mice that overexpressed indoleamine 2,3-dioxygenase (IDO), a tryptophan-catalyzing enzyme that allows tumor cells to evade host immune activity [516]. The compound was able to block IDO both in vivo and in vitro [516]. Aspirin, often used for CVD, has also been shown to prevent the onset of gastrointestinal cancers in a phase 3 clinical trial [517] through its ability to upregulate SIRT1 expression [325]. Pterostilbene (PTE), another popular natural SIRT1 activator, has been shown to lessen inflammatory responses and be neuroprotective via reduction of neuronal cell apoptosis and oxidative stress [518]. PTE demonstrated prevention of cancer development and even metastasis in wildtype p53 prostate cancer cells that were stopped from progressing past the G1 phase of the cell cycle following p53 and p21 upregulation. Additionally, prostate cancer cells lacking p53 underwent apoptosis upon pterostilbene administration [519]. PTE also reduced cellular inflammation and oxidative stress through antioxidant amplification within male BALB/c mice injected with azoxymethane, a common inducer of colon cancer, used in modeling colon carcinogenesis [520]. The benefits of PTE are not limited to prostate and colon cancers, as the compound has been linked with anticancer effects in many other cancers, such as lung, cervical, and hepatocellular carcinomas [521]. There is great potential for these drugs, and several of them are undergoing clinical trials for both cancer and aging-related diseases (see Supplementary File S4 and Table 4).

## 8. Biomarkers for Telomere Attrition in Cancer and Aging

Telomere length serves as a promising prognostic biomarker for cancer. According to a 2020 review paper, there is an association between shorter leukocyte telomere length (LTL) and higher overall mortality rates for cancer patients, except for those with secondary thyroid cancers [522]. Specifically, children who had undergone chest radiotherapy or abdominal/pelvic radiotherapy presented shorter LTL post-treatment than children without cancer (control group) [522]. Slightly longer LTL was present at the time of cancer diagnosis in childhood, which is consistent with telomere elongation in few cancers; however, childhood cancer survivors have significantly lower LTL due to higher telomere attrition rates throughout their young adult and adult life [522,523]. This finding was observed in children affected by several sarcomas, neuroblastomas, and acute lymphoblastic leukemia [524], and it is important to note that LTL is primarily used as a marker within hematological cancers. Contrarily, it has been shown to predict all-cancer mortality across a large cohort of 64,637 individuals [525]. This accelerated telomere attrition can be due to the cytotoxic effects of cancer treatments like chemotherapy and radiation, which are known to induce DNA damage and oxidative stress [522,523]. Relapsed cancer is also significantly associated with shorter relative telomere length for hematologic malignancies [526]. Nonetheless, more research is needed to empirically determine the sensitivity of LTL as a biomarker for healthy survivorship for childhood cancer treatment [522]. Interestingly, numerous reports have highlighted an association with shorter leukocyte telomere length and increased diagnosis of cardiomyopathy, one of the major age-related pathologies [527,528,529], highlighting the potential of LTL as a biomarker for cancer and aging.

Several biomarkers can indirectly indicate the severity of telomere attrition and the potential for carcinogenesis and progression. For example, 8-oxoguanine is a common DNA lesion that is caused by ROS, which serves as a large contributor to senescence and age-related diseases [190,241,454]. 8-oxoguanine levels can serve as a biomarker for the DNA damage response and may indirectly indicate accelerated telomere shortening [530]. Overall, 8-oxo is a sensitive marker for DNA damage, which might lead to telomere shortening, but it is not a specific marker for telomere attrition. Therefore, biomarkers such as gamma-H2AX signals may serve as a more appropriate sign of senescence as they mark senescent cells within culture and tissues [531]. Telomere Dysfunction-Induced Foci (TIF) analysis is a more telomere-specific tool used in vitro or in tissue sections to detect gamma-H2AX signals and 53BP1 via the use of one or more antibodies [532]. TIF can also be used to detect telomere damage via antibodies directed toward components of the shelterin complex, such as TRF2 [532]. However, this approach does not provide information about the length of the telomere; instead, it only elucidates telomere damage [533]. Interphase Quantitative Fluorescence In Situ Hybridization (Q-FISH) is useful for measuring telomere length for in vitro cells, where a microscope is used to visualize telomere fluorescence intensity after hybridization with a fluorescent peptide nucleic acid (PNA) telomeric repeat probe [534]. One disadvantage of Interphase Q-FISH is that some intense signals might represent several telomeres clustered closely [534]. Single TElomere Length Analysis (STELA), a PCR-based technique, is another approach to measuring telomere length, which is more tailored to analyzing the individual telomeres of single chromosomes [535].

C-reactive protein level is a biomarker for acute inflammation and is a good predictor of health spans due to their high sensitivity to inflammation [536,537,538]. Senescence-associated beta-galactosidase (SA-B-Gal) [539,540] and senescence-associated heterochromatic foci (SAHF) [53] are two sensitive biomarkers for senescence but are not specific to any age-related disease [539]. Therefore, these two biomarkers need to be considered with other specific biomarkers to help uncover particular age-related diseases the individual is more likely to develop. For example, homocysteine is an example of a more specific biomarker for CVD as it increases the rates of telomere attrition, accelerating endothelial cell senescence [455]. Advanced glycation end products are more connected to T2DM development [541], while TPP1 stability is a biomarker for telomere dysfunction in COPD [375].

## 9. Conclusions

**Figure 9:**
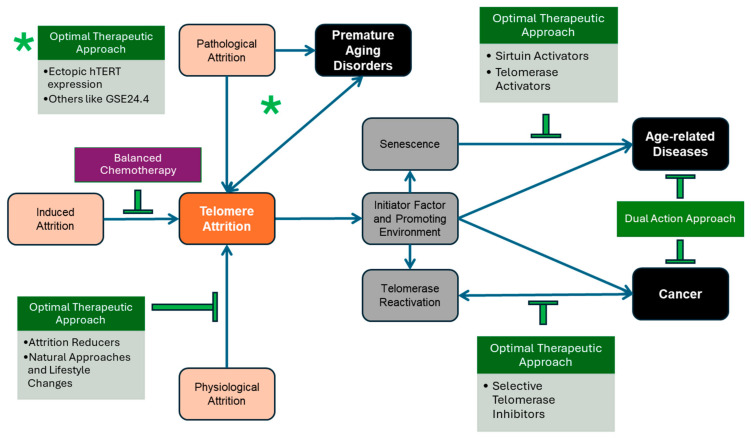
Landscape of the OTA. The usage of OTA to tackle cancer, aging, and premature aging disorders. * OTA for Premature Aging Disorders. Green arrows represent inhibition arrows. Figure colors are used for contrast only and do not represent any meaning beyond showing the different elements of the figure.

Telomere maintenance plays a vital role in cancer immortality, mainly through telomerase reactivation and ALT, demonstrating the importance of telomere maintenance in carcinogenesis and disease progression. Telomere attrition promotes senescence, leading to various age-related diseases, and it also induces the development of premature aging disorders. While premature aging disorders are relatively rare, having a mutational component, developing chronic age-related diseases like CVD and neurodegeneration are more common. The field of telomere maintenance holds great potential for understanding and developing better approaches that can counteract telomere attrition to combat aging (Figure 9), including the decrease in oxidative stress and inflammation. It also warrants the need for more information to find a balance between critically short telomeres rejuvenation without elongating those of cancer cells.

Our paper provides an aggregation of updated knowledge and direction to support this endeavor; however, it is bound by the limitations of our scope. For example, our review does not include papers that discuss other traditional modes of therapy, such as immunotherapy and radiation, which might hold the potential to serve as OTA. This review does not cover all current relevant studies comprehensively, which may lead to an incomplete depiction of the status quo of the field. Moreover, we do not include a formal assessment of the quality of the studies included, which can affect reliability. After examining ClinicalTrials.gov, we have identified a limited number of clinical trials that focus primarily on telomere biology. Our paper hopes to support the advancement of clinical research for further investigation of the safety and efficacy of these promising findings, especially ones that follow the principles of OTA.

We believe future clinical practices should aim to balance cancer therapy to avoid aging acceleration and vice versa. Selective telomere inhibitors, such as Imetelstat, are promising therapies that seem to achieve this balance. While the efficacy of telomerase inhibitors might be less than traditional approaches for treating certain cancers, their use in combination therapy might mitigate the impact of chemotherapy on telomere attrition for non-cancerous cells. Regarding aging, the addition of sirtuin activators and other discussed approaches have the potential to promote healthy aging without increasing the chance of carcinogenesis. Telomere biology impacts the entire human body; therefore, we argue that the expansion of telomere research to encompass a broader range of cancer types and age-related diseases will significantly enhance our understanding of molecular pathways and their clinical significance, leading to improved therapeutics.

## Figures and Tables

**Table 1:** Survey of genetic approaches for cancer.

Genetic Approach	Mechanism of Action as Described in Source	Source
Small Interfering RNA (siRNA) for TERT and TERC	Binds to its complementary telomerase mRNA (knockdown).	[223,224,225,226]
Dominant-negative Telomerase (dnTERT)	Inhibits the activity of telomerase and causes apoptosis.	[227,228]
AAV-based Gene Therapy (TERT, TERC, et al.)	Induces the transient expression of telomerase or components of the shelterin complex with no increased cancer incidence.	[229,230,231,232,233]
Antisense Oligonucleotides (ASO) for TERT and TERC	Selectively weakens TERC expression and inhibits TERT activity in TERT-positive cancer cells while preserving telomere length.	[169,220,221]
Oncolytic Adenovirus	Cancer-specific adenoviruses can suppress tumor growth for up to 9 or 30 days through pTERT.	[215,216,217,218]
CRISPR/Cas9 targeting TERT	Targets the 3′-UTR region to promote senescence and apoptosis, shown to weaken telomerase activity	[212,213]
Adenoviral shRNA Vector (ad-shRNA) for TERC and TERT	Decreases the target’s mRNA expression, with Ad-shTERC inhibiting telomerase activity more than Ad-shTERT.	[234]

A survey of contemporary genetic approaches that may contribute to advancements in cancer research and treatment. Refer to Supplementary File S3 for the complete table.

**Table 4:** Clinical trials for dual-action drugs.

Name (Alternative Name)	NCT Identifier	Clinical Trial Name	Study Start (Actual) to Study Completion (Actual/Estimated)	Phase and Status
Rapamycin (Sirolimus)	NCT04488601	Participatory Evaluation (of) Aging (With) Rapamycin (for) Longevity Study (PEARL)	2020–2023	Phase 2: Completed
Rapamycin (Sirolimus)	NCT05840510	Adagrasib in Combination With Nab-Sirolimus in Patients With Advanced Solid Tumors and Non-Small Cell Lung Cancer With a KRAS G12C Mutation (KRYSTAL -19)	2023–2026	Phase 1: Active, not recruiting
Rapamycin (Sirolimus)	NCT04375813	Trial of Encapsulated Rapamycin (eRapa) for Bladder Cancer Prevention	2021–2026	Phase 2: Recruiting
Berberine	NCT03770325	A Mechanistic Randomized Controlled Trial on the Cardiovascular Effect of Berberine	2019–2020	Phase 2/3: Completed
Berberine	NCT03281096	A Research of Berberine Hydrochloride to Prevent Colorectal Adenomas in Patients With Previous Colorectal Cancer	2017–2021	Phase 2/3: Completed
Resveratrol	NCT02523274	Resveratrol and Exercise to Treat Functional Limitations in Late Life	2016–2019	Phase 2: Completed
Resveratrol	NCT03253913	Resveratrol and Sirolimus in Lymphangioleiomyomatosis Trial (RESULT)	2018–2022	Phase 2: Completed
Statins	NCT02968810	Simvastatin in Preventing Liver Cancer in Patients With Liver Cirrhosis	2017–2024	Phase 2: Active, not recruiting
Statins	NCT04418089	Simvastatin Effect in Combination With Neoadjuvant Chemotherapy to Clinical Response and Tumor-Free Margin in Locally Advanced Breast Cancer	2018–2019	Phase 2: Completed
Statins	NCT02787590	Simvastatin as a Neuroprotective Treatment for Moderate Parkinson’s Disease (PD STAT)	2016–2020	Phase 2: Completed

Some clinical trials from 2014-present that show the potential of such drugs as therapeutic approaches for cancer and aging. Data were collected from ClinicalTrials.gov (accessed from 1 October 2024 to 10 October 2024). Refer to Supplementary File S4 for the complete table.

## Data Availability

All the data are presented within the manuscript files associated with the manuscript text.

## Supplementary Materials

The following supporting information can be downloaded at https://www.mdpi.com/article/10.3390/cancers17020257/s1.
